# Mechanistic Insights Into Functional Innovations of Dammarenediol‐II Synthase in *Panax ginseng*

**DOI:** 10.1111/pbi.70299

**Published:** 2025-10-09

**Authors:** Lei Feng, Yan Zhao, Dongju Fu, Xia Li, Bing Hao, Yucheng Zhao, Xiangyu Liu, Guisheng Xiang, Zihan Yang, Fengling Tan, Meiyu Duan, Hanyu Fu, Bolin Wu, Simei He, Yina Wang, Geng Chen, Shuangyan Zhang, Chuyi Wang, Wanling Song, Yuanhong Fan, Guanghui Zhang, Shengchao Yang

**Affiliations:** ^1^ College of Agronomy and Biotechnology Yunnan Agricultural University Kunming China; ^2^ National‐Local Joint Engineering Research Center on Gemplasm Innovation & Utilization of Chinese Medicinal Materials in Southwest, The Key Laboratory of Medicinal Plant Biology of Yunnan Province Kunming Yunnan China; ^3^ Yunnan Characteristic Plant Extraction Laboratory Co. Ltd. Yunnan China; ^4^ Department of Resources Science of Traditional Chinese Medicines China Pharmaceutical University Nanjing China; ^5^ Honghe University Honghe Yunnan China

**Keywords:** amyrin synthases, dammarenediol‐II synthetases, evolution, *Panax ginseng*

Plant secondary metabolites, particularly triterpenoids, have significant bioactivities and are highly valued (De La Peña et al. 2023; Salmon et al. 2016; Zhao et al. 2023). Oxidosqualene cyclases (OSCs) catalyse the conversion of 2,3‐oxidosqualene (OS) into dammarane‐type and pentacyclic triterpenoid skeletons (Abe 2007; Salmon et al. 2016; Gao et al. 2024; Jo et al. 2025). To date, over 170 OSC genes have been identified, with functional diversity mainly from key members *β*‐amyrin synthase (*β*‐AS), dammaradienol synthase (DDS) and cycloartenol synthase (CAS) (Chen et al. 2021). Notably, despite sharing a common dammarenium cation intermediate (Figure 1a), *β*‐AS and DDS produce structurally distinct products: *β‐*AS synthesises pentacyclic *β*‐amyrin, whereas DDS generates tetracyclic dammarenediol‐II (DM‐II) (Salmon et al. 2016; Chen et al. 2021). Gene duplication and neofunctionalisation, like *Panax* DDS from *β*‐AS evolution, shaped OSC family diversification (Zhang et al. 2023), but the molecular mechanisms of product specificity are still unclear, challenging triterpenoid metabolic evolution understanding and enzyme engineering advancement.

**FIGURE 1:**
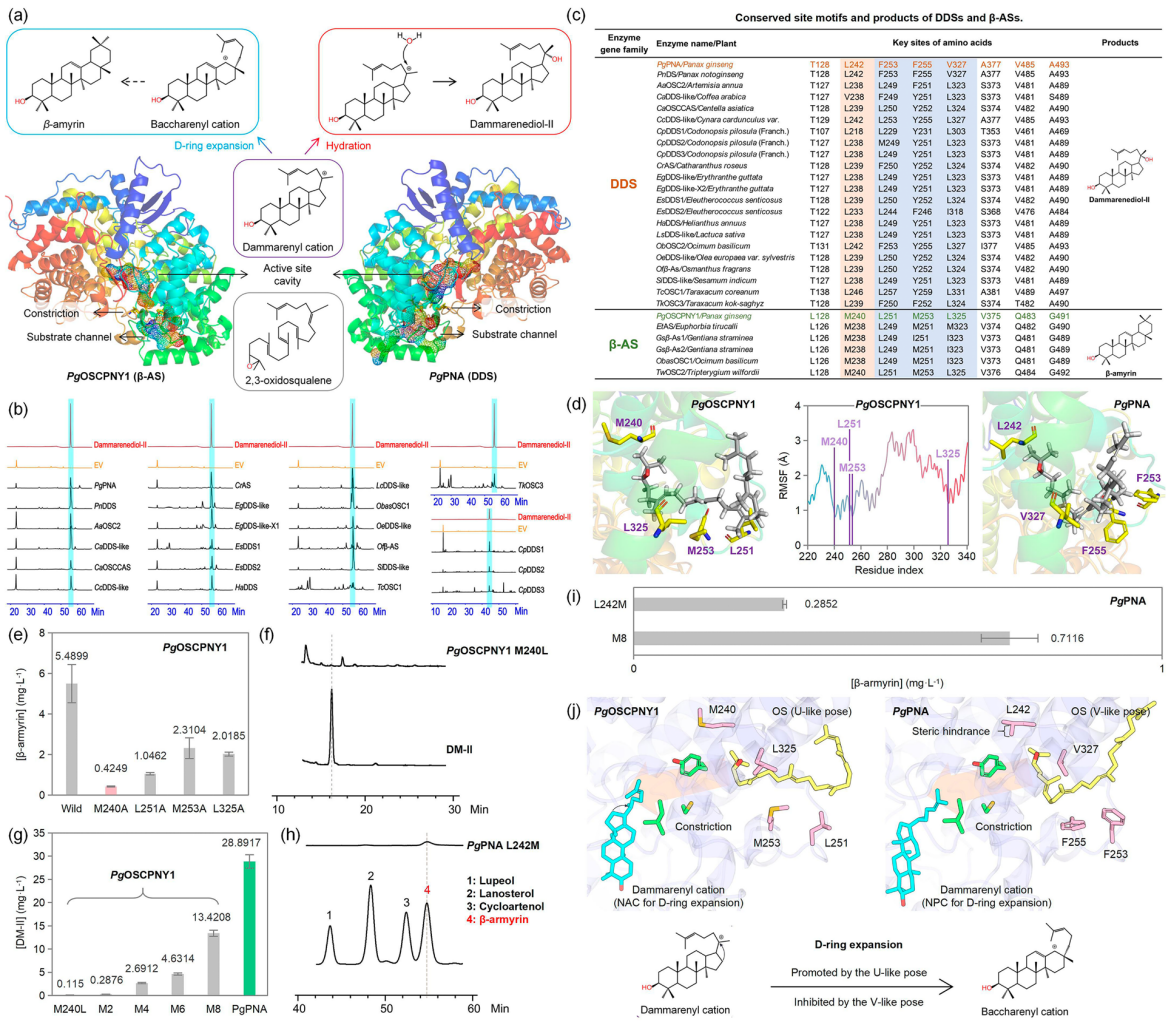
(a) Cyclisation reactions carried out by the *β*‐amyrin synthase and dammarenediol‐II synthase from *P. ginseng* . The dashed arrow indicates the omission of multiple cation intermediates. (b) HPLC chromatograms of the functional characterisation of 22 DDS genes. (c) Sequence alignments between 22 DDSs and 6 *β*‐ASs. (d) OS binding poses and root mean square fluctuation (RMSF) analysis of *Pg*OSCPNY1‐OS complex. (e) The concentration of *β*‐amyrin produced by *Pg*OSCPNY1 and its alanine mutants. (f) Detection of DM‐II content using HPLC in *Pg*OSCPNY1^M240L^. (g) The concentration of DM‐II produced by *Pg*OSCPNY1^M240L^, combined mutants and wild‐type *Pg*PNA. (h) Detection of DM‐II content using HPLC in *Pg*PNA^L242M^. (i) The concentration of *β*‐amyrin produced by *Pg*PNA^L242M^ and *Pg*PNA^M8^. (j) Differential mechanisms of substrate conformational preorganisation. Error bars represent standard deviation SD (*n* = 3 independent samples).

The DDS gene family of *P. ginseng* originated from the novel functionalisation of *β*‐AS and other mTTS genes after the whole‐genome replication event in Araliaceae (Yang et al. 2023). To explore the evolutionary and functional aspects of DDSs, we retrieved 39 putative DDS‐like genes from NCBI and conducted phylogenetic analysis with other OSC genes. The phylogenetic tree showed DDS and *β*‐AS genes formed distinct clusters (Figure S1), indicating significant sequence differences due to long‐term functional domain mutations and natural selection. We further performed heterologous expression of the 39 putative DDS‐like genes in *Saccharomyces cerevisiae* and characterised their enzymatic activities. Functional screening showed 22 out of 39 candidate DDSs had catalytic activity, with HPLC and LC–MS confirming they only produced DM‐II, without *β*‐amyrin (Figure 1b).

To explore the relationship between the function and sequence of *β*‐ASs and DDSs, we aligned 6 *β*‐ASs and 22 DDSs sequences. The results revealed that 8 key residues (L128, M240, L251, M253, L325, V375, Q483 and G491 in *Pg*OSCPNY1) were highly conserved within the same functional group (Figure 1c). The differences in these residues may explain functional differences between DDSs and *β*‐ASs. Docking and dynamics simulations offer further insights (Figure 1d). In *Pg*OSCPNY1, M240, L251, M253 and L325 are in the substrate channel, forming strong hydrophobic interactions with OS. They exhibited low flexibility and high stability during simulations, crucial for OS positioning. However, a constriction site separates the substrate from the active‐site cavity (Figure 1a). Substrate passage may be facilitated by alterations in the side chain conformations of Y266, C262 and I556 (Thoma et al. 2004). A similar pattern exists in DDSs, distinguished by variations in these key residues. To pinpoint the hotspot residue in *Pg*OSCPNY1, we performed alanine mutagenesis on these conserved sites (M240, L251, M253, and L325) in the substrate channel. Compared to wild‐type *Pg*OSCPNY1, mutating these key residues reduced *β*‐amyrin yield, with the *Pg*OSCPNY1^M240A^ showing the most significant decrease (Figure 1e). This highlights that M240 is a hotspot for enzyme activity. We substituted M240 with Leu found in DDSs via mutagenesis, showing this change gave *Pg*OSCPNY1 DDS‐like capabilities, proving M240L variation is key to DDS functional innovation in *P. ginseng* (Figure 1f).

However, the *Pg*OSCPNY1^M240L^ variant produced DM‐II at a concentration of only 0.4% compared with that of wild‐type *Pg*PNA (Figure 1g). Using the equivalent sites in DDSs as a template, we designed a series of combined mutants in *Pg*OSCPNY1 (M2: M240L/Q483V; M4: M240L/L251F/M253F/Q483V; M6: M240L/L251F/M253F/L325V/Q483V/G491A; M8: L128T/M240L/L251F/M253F/L325V/V375A/Q483V/G491A) to reconstruct the activity and function of DDSs. The site‐directed mutagenesis results confirmed DM‐II production increase from M240L to the M8 mutants (Figure 1g). The M8 mutant reached 46.45% of catalytic efficiency in wild‐type *Pg*PNA. This finding indicates that the high efficiency of DDSs largely originates from concerted variations at these conserved sites in *Pg*OSCPNY1.

*Pg*PNA^L242M^ was designed as a reverse mutant, indicating a *β*‐AS‐like function (Figure 1h). The *β*‐amyrin yield of *P*gPNA^L242M^ reached only 5.19% of that of *Pg*OSCPNY1 (Figure 1h). Combined reverse design of *Pg*PNA created *Pg*PNA^M8^ (T128L/L242M/F253L/F255M/V327L/A377V/V485Q/A493G) via mutagenesis. Its *β*‐amyrin yield reached 0.7116 mg·L^−1^, a 149.51% increase over *Pg*PNA^L242M^ (Figure 1i).

Further analysis of the OS and the dammarenyl cation binding patterns (Figure 1j) revealed that in *Pg*OSCPNY1, the steric hindrance of L251, M253 and L325 caused OS to adopt a ‘U‐like’ pose. In *Pg*PNA, the corresponding residues F253, F255 and V327 promoted the formation of a ‘V‐like’ pose. The former produces a near‐attack conformation (NAC) promoting D‐ring expansion of the dammarenyl cation, while the latter corresponds to a non‐productive conformation (NPC), causing its hydration (Figure S6). The steep increase in the DM‐II concentration produced by *Pg*OSCPNY1^M8^ revealed a significant increase in the DDS‐like function via the ‘V‐like’ pattern. In contrast, *Pg*PNA^M8^ exhibited a catalytic efficiency that was 149.51% greater than that of *Pg*PNA^L242M^, revealing the profound impact of the ‘U‐like’ structural motif on *β*‐AS‐like function.

We propose that the *β*‐AS and DDS enzymes in *P. ginseng* produce *β*‐amyrin and DM‐II via preorganised conformations that either support or hinder the D‐ring expansion of the dammarenyl cation (Figure 1j). The substrate preorganisation initiates within the substrate channel and is further refined by the steric hindrance of residues M240 and L242. In *Pg*OSCPNY1^M240L^ and *Pg*PNA^L242M^, owing to the absence of a corresponding transformation of the substrate pose, a precipitous decrease in activity was observed. Conversely, the combined mutants of *Pg*OSCPNY1 harbouring L251F/M253F/L325V and the *Pg*PNA^M8^ mutant harbouring F253L/F255M/V327L resulted in a directed transition of the pose, thereby significantly increasing the new functions. Mutations at other key sites, including L128T, V375A and Q483V, can enhance the stability and thus improve the catalytic efficiency of the novel enzyme *Pg*OSCPNY1^M240L^, whereas the G491A mutation exhibits the opposite effect (Figure S7).

Multiple sequence alignment of various Araliaceae DDSs and *β*‐ASs revealed *Eleutherococcus senticosus* and *Panax* (including *P. ginseng* and *P*. *notoginseng*) DDSs share the conformation fine‐tuning mechanism of M240L (*Pg*OSCPNY1) and stability‐related mutations L128T/Q483V/G491A (Figure S8). However, *E. senticosus* lacks the ‘V‐like’ motif transformation, with activity significantly lower than *Panax* DDSs (Figure S9), further confirming the critical role of this motif in DM‐II production. The high conservation of T128, L242, V485 and A493 (*Pg*PNA) across DDSs of different species reveals convergent evolution in DDSs (Figure 1c). In contrast, the unique ‘V‐like’ motif represents a lineage‐specific innovation in *Panax*.

Taken together, *P. ginseng* DDS innovations arise from cooperative *β*‐AS mutations: four key variations shape and fine‐tune novel substrate preorganisation to regulate *β*‐amyrin/DM‐II selectivity, while other mutations affect novel enzyme stability differently. Their conservation and divergence across DDSs reveal widespread convergent evolution and *Panax*‐specific innovations.

## Conflicts of Interest

The authors declare no conflicts of interest.

## Supporting information


**Data S1:** pbi70299‐sup‐0001‐DataS1.docx.

## Data Availability

The data that supports the findings of this study are available in the Supporting Information of this article.
