# Supplementary material for: Mechanistic Insights Into Functional Innovations of Dammarenediol‐II Synthase in Panax ginseng
Source: Plant Biotechnol J. 2025 Oct 9;24(3):1061–3. doi: 10.1111/pbi.70299 (PMC12946519; doi:10.1111/pbi.70299)
Supplement: Supplementary file 1 — Data S1: pbi70299‐sup‐0001‐DataS1.docx. [file PBI-24-1061-s001.docx]

**Supporting information**

**Figure S1** Phylogenetic analysis of OSCs.

**Figure S2** EIC and M/Z determination for the fermentation product, DM-II, of the 22 DDSs via LC‒MS.

**Figure S3** *β*-amyrin content in wild-type and alanine mutant-type *Pg*OSCPNY1.

**Figure S4** Dammarenediol-II content in mutant *Pg*OSCPNY1 and wild-type *Pg*PNA.

**Figure S5** *β*-amyrin content in mutant-type *Pg*PNA.

**Figure S6** Zoom of the active site showing, in *Pg*OSCPNY1 and *Pg*PNA, the NAC facilitating and the NPC inhibiting D-ring expansion.

**Figure S7** The effects of L128T, V375A, Q483V, and G491A mutations on the stability and catalytic efficiency of the novel enzyme *Pg*OSCPNY1^M240L^.

**Figure S8** Sequence alignments between DDSs and β-ASs from Araliaceae, including *Panax ginseng*, *Panax notoginseng*, and *Eleutherococcus senticosus*.

**Figure S9** Dammarenediol-II content in Araliaceae DDSs.

**Table S1** Primers used for cloning.

**Table S2** YPD medium formula.

**Table S3** SC-Ura medium formula.

**Table S4-S6** HPLC procedures for products.

**Table S7** LC-MS procedures for products.

**Data S1** Gene sequences of enzymes.

Supplemental Tables and Figures for

Mechanistic Insights into Functional Innovations of Dammarenediol-II Synthase in *Panax ginseng*

**
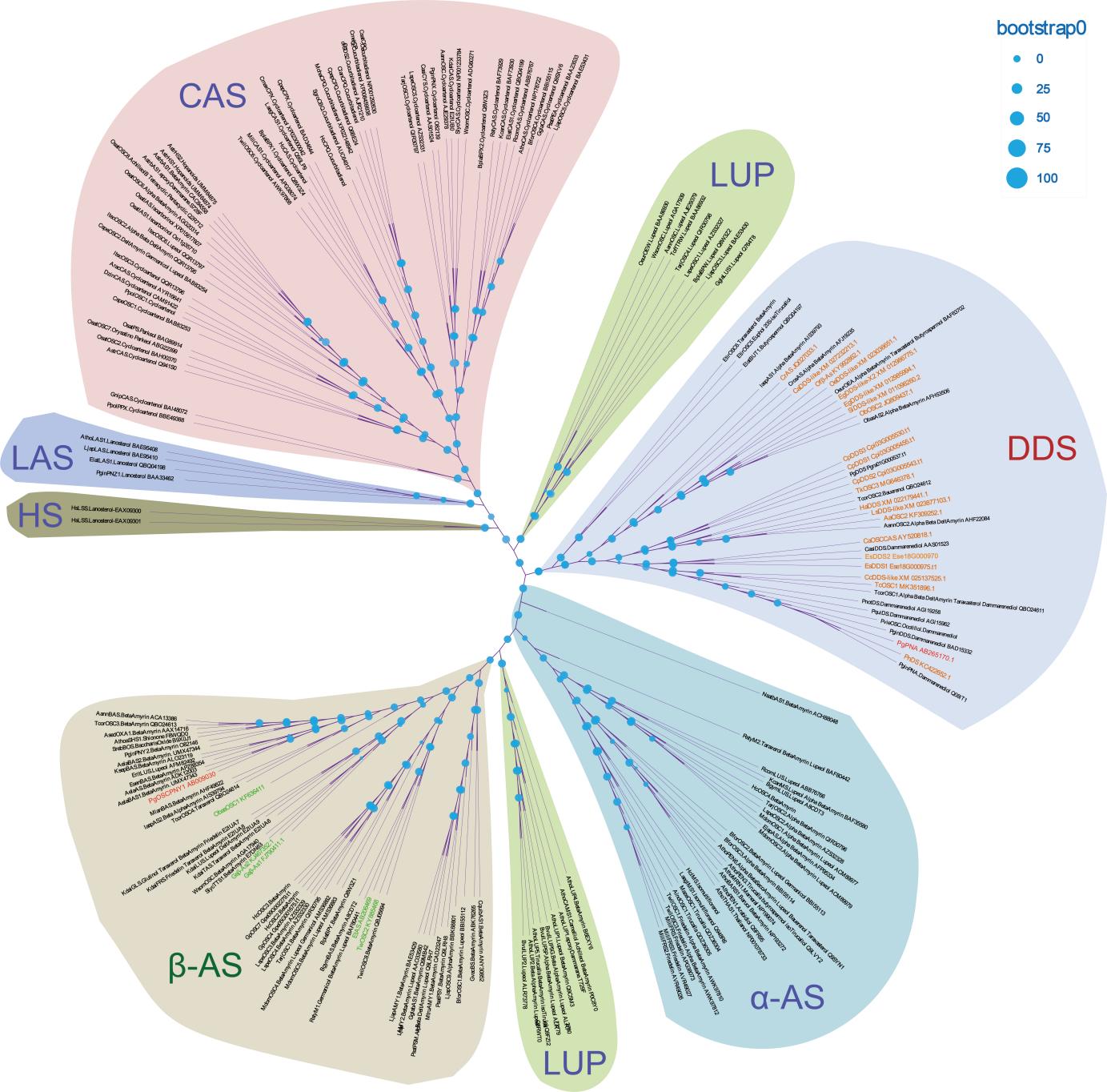
**

**Figure S1** Phylogenetic analysis of OSCs.

The orange and red items in DDS represent all 22 enzymes that produce DM-II and have no production of *β*-amyrin. The green and red items in *β*-AS represent all 6 enzymes that produce *β*-amyrin and have no production of DM-II. The DDS and *β*-AS enzymes of *P. ginseng* are marked in red.

**
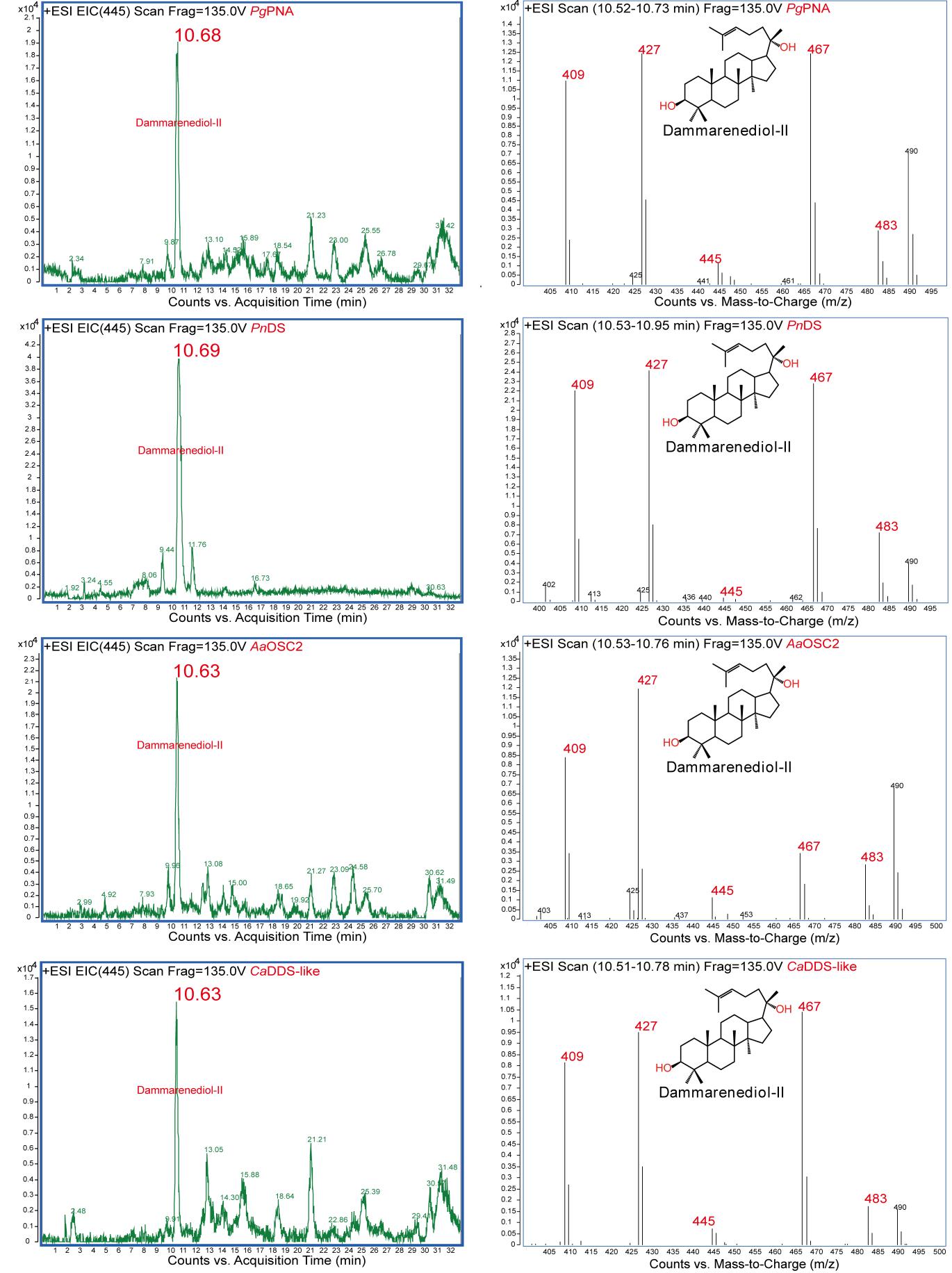
**

**
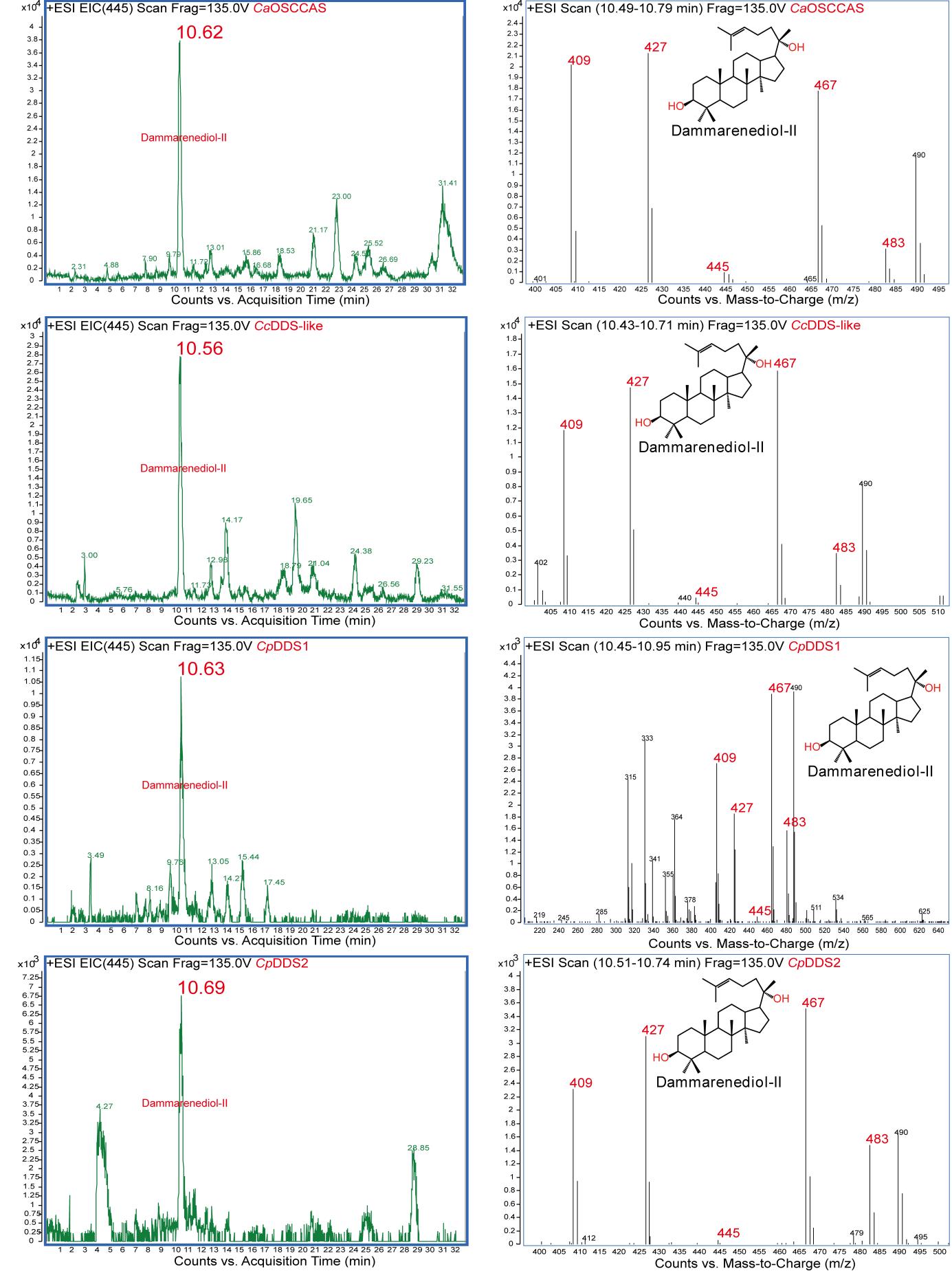
**

**
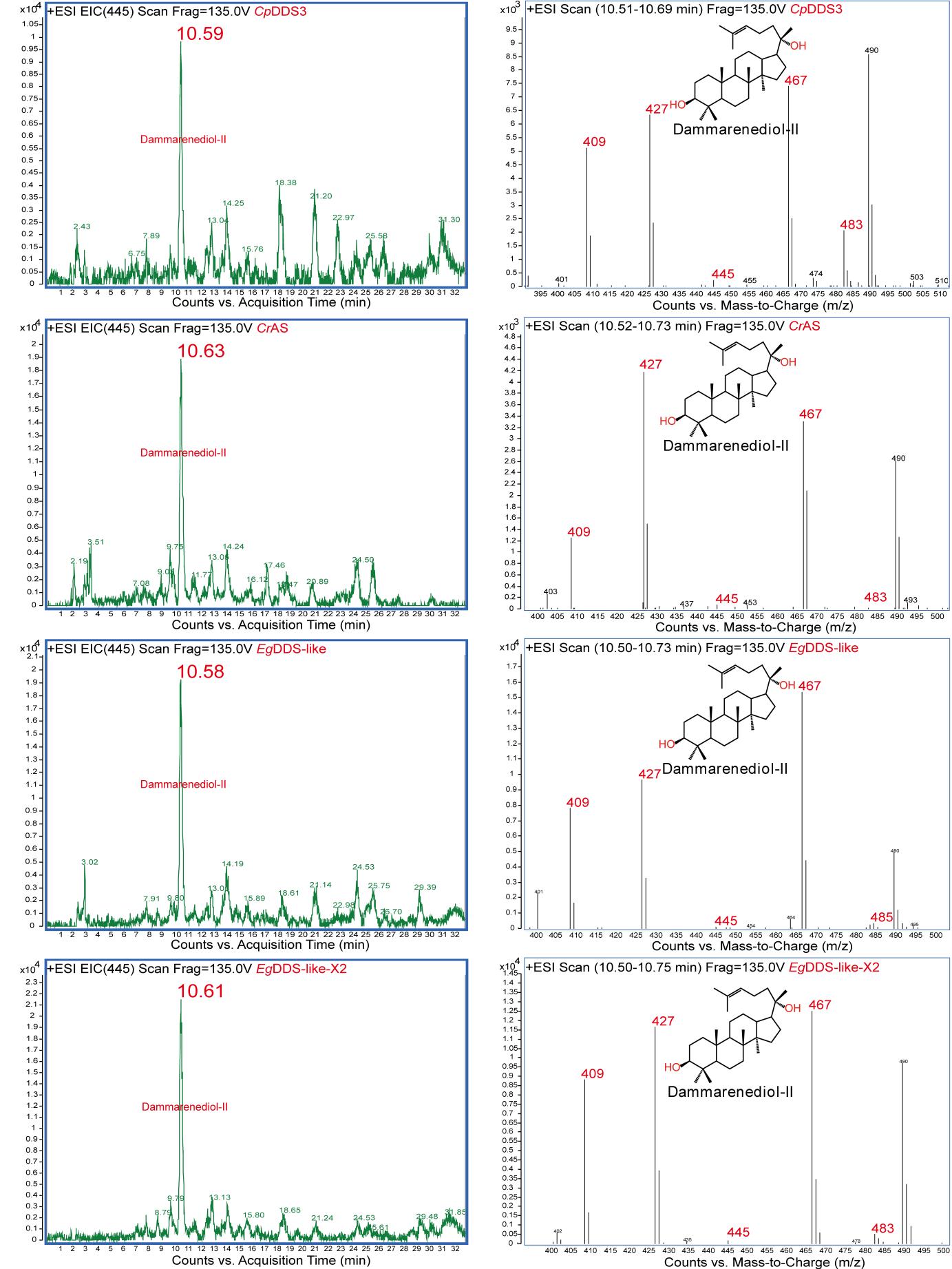
**

**
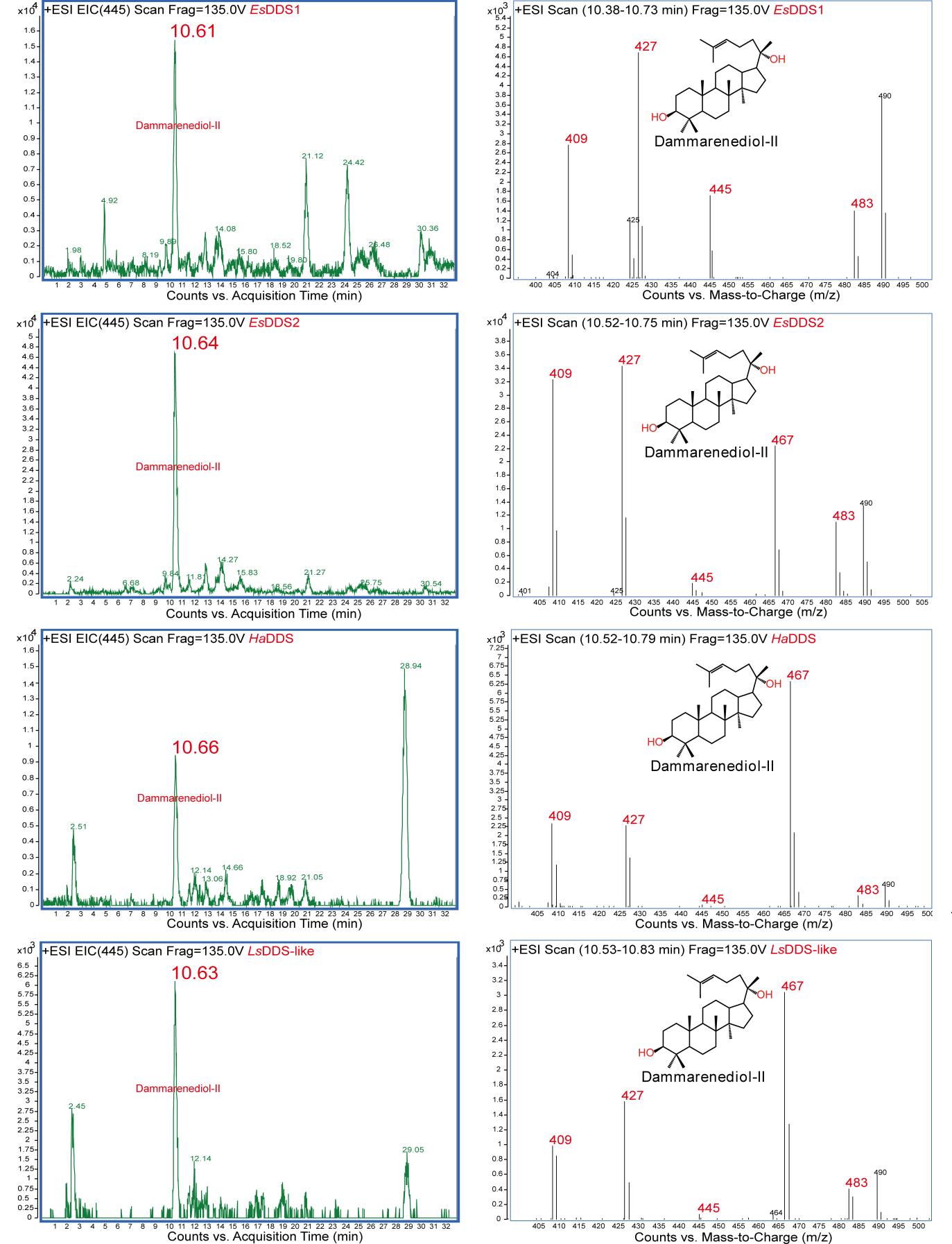
**

**
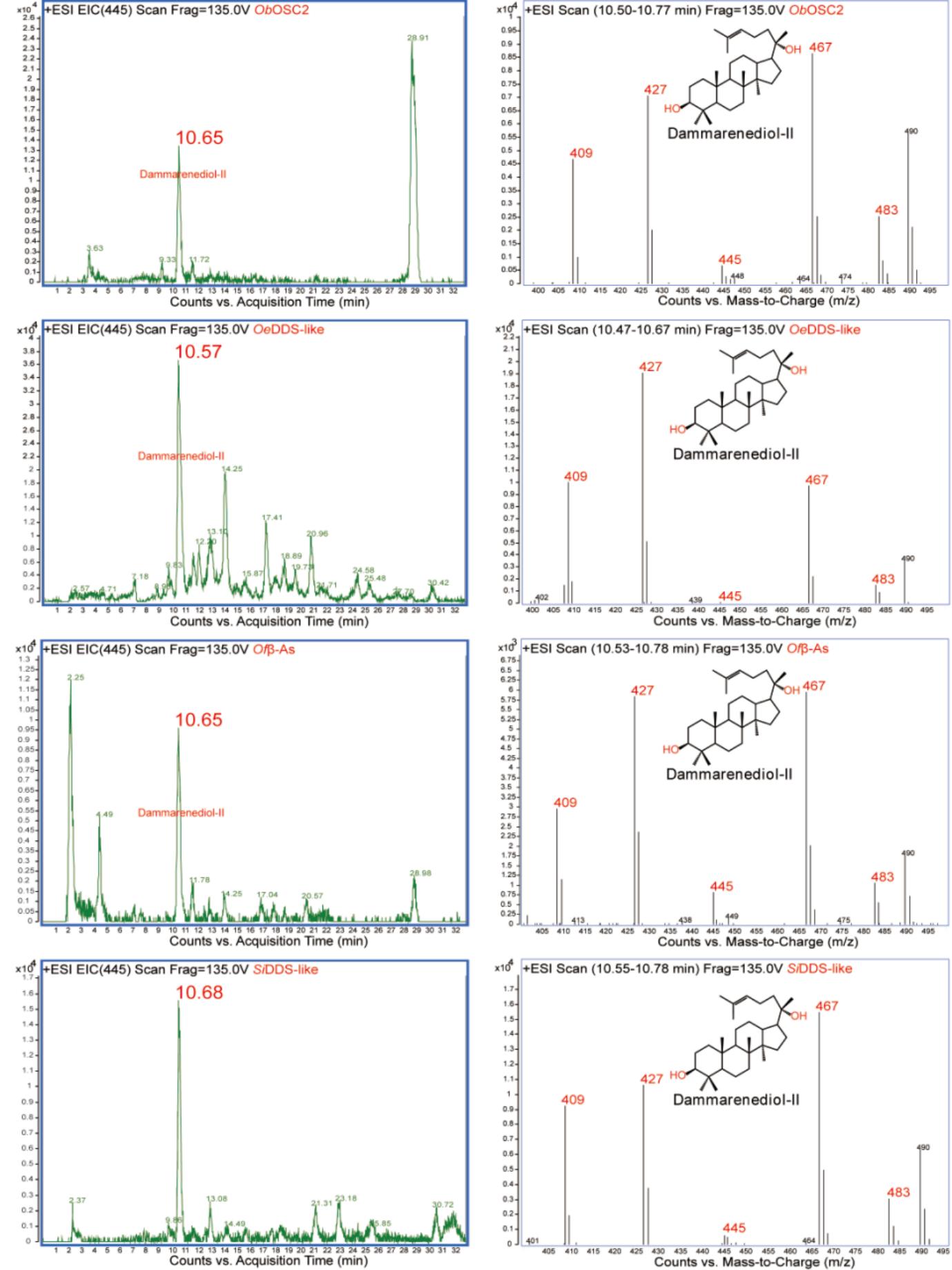
**

**
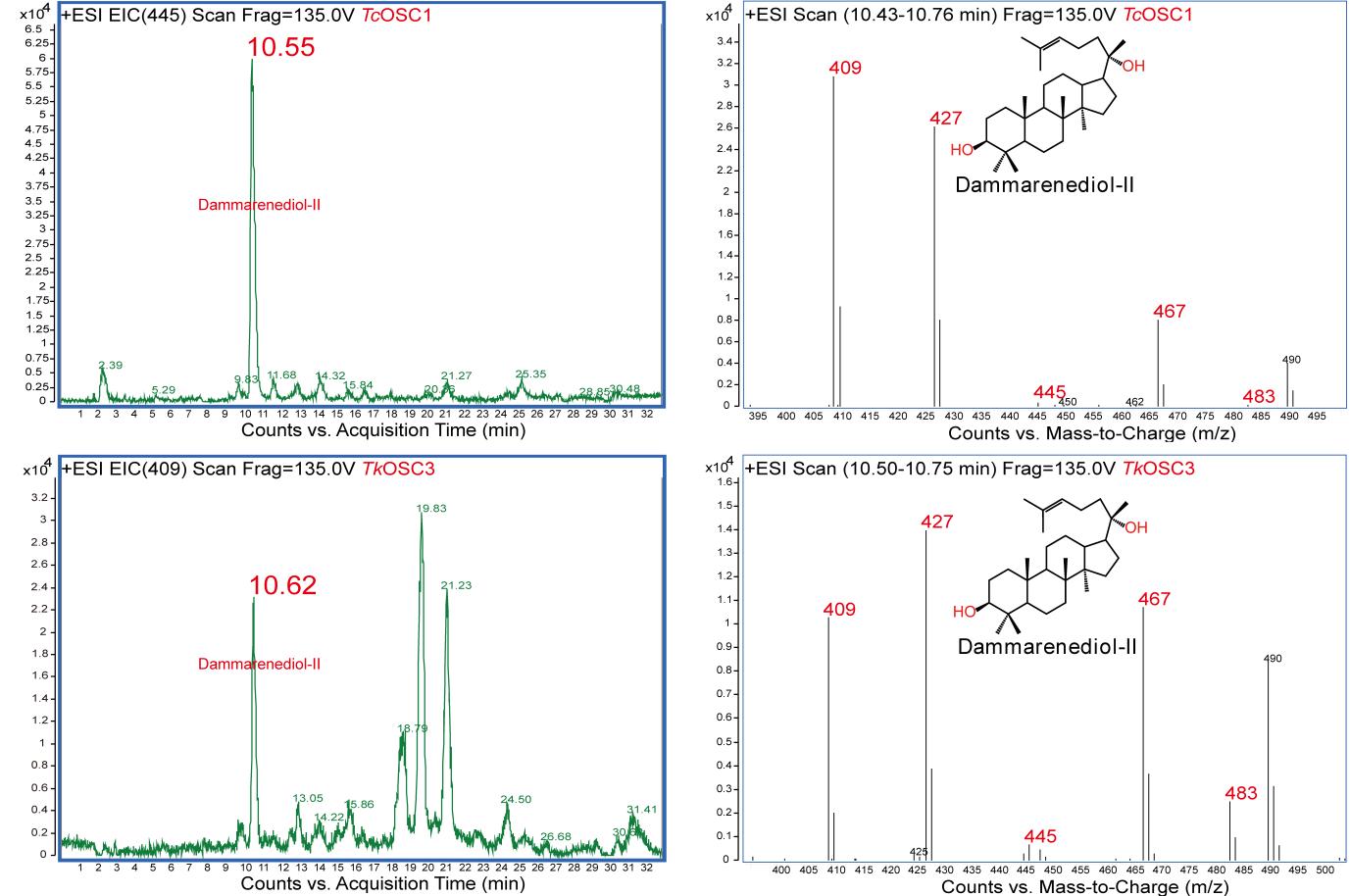
**

**Figure S2** EIC and M/Z determination for the fermentation product, DM-II, of the 22 DDSs via LC‒MS.


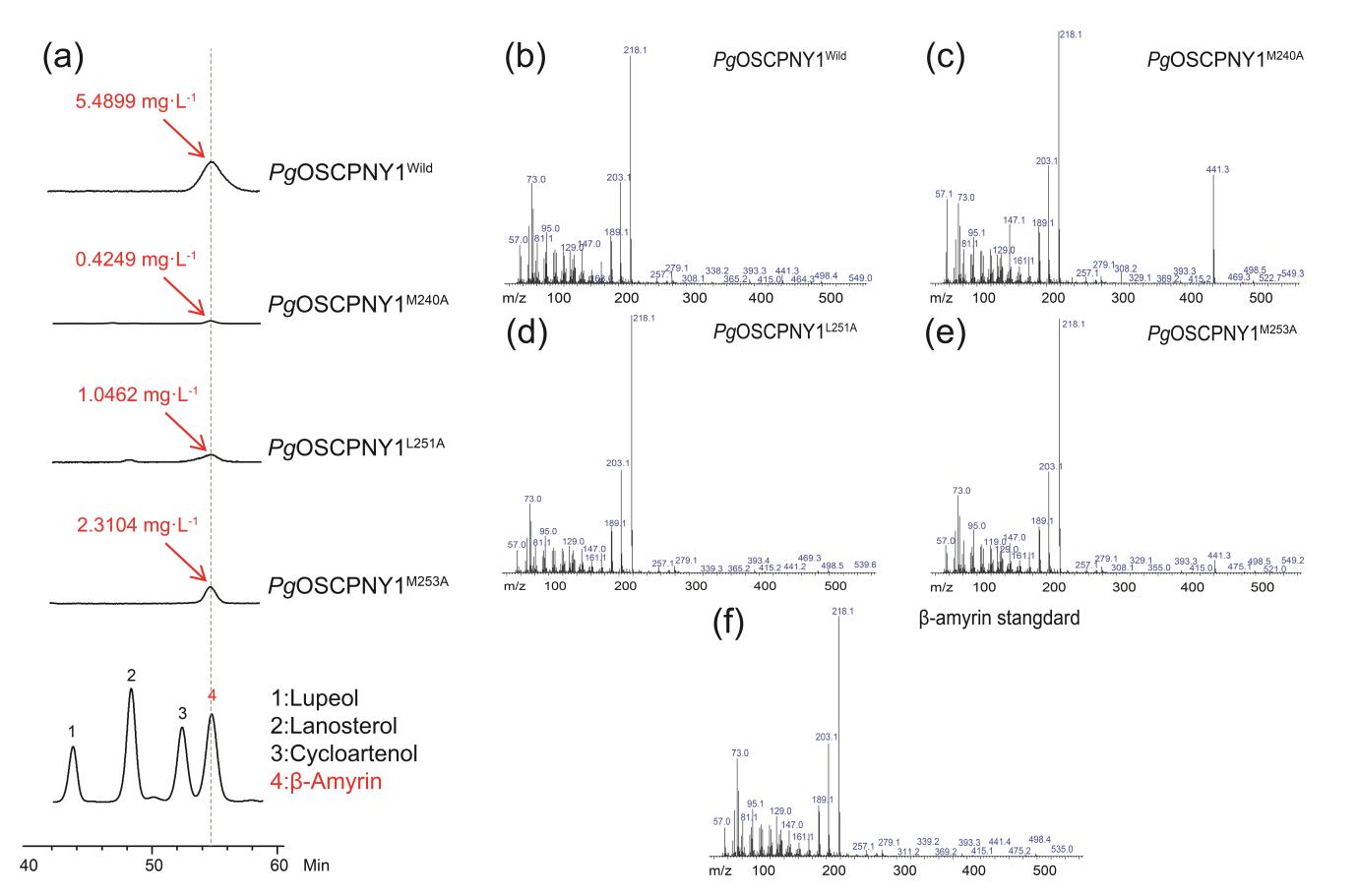
**Figure S3** *β*-amyrin content in wild-type and alanine mutant-type *Pg*OSCPNY1.

(a) Detection of *β*-amyrin content using HPLC. (b-f) GC-MS analysis of *β*-amyrin (trimethylsilane derivatives).


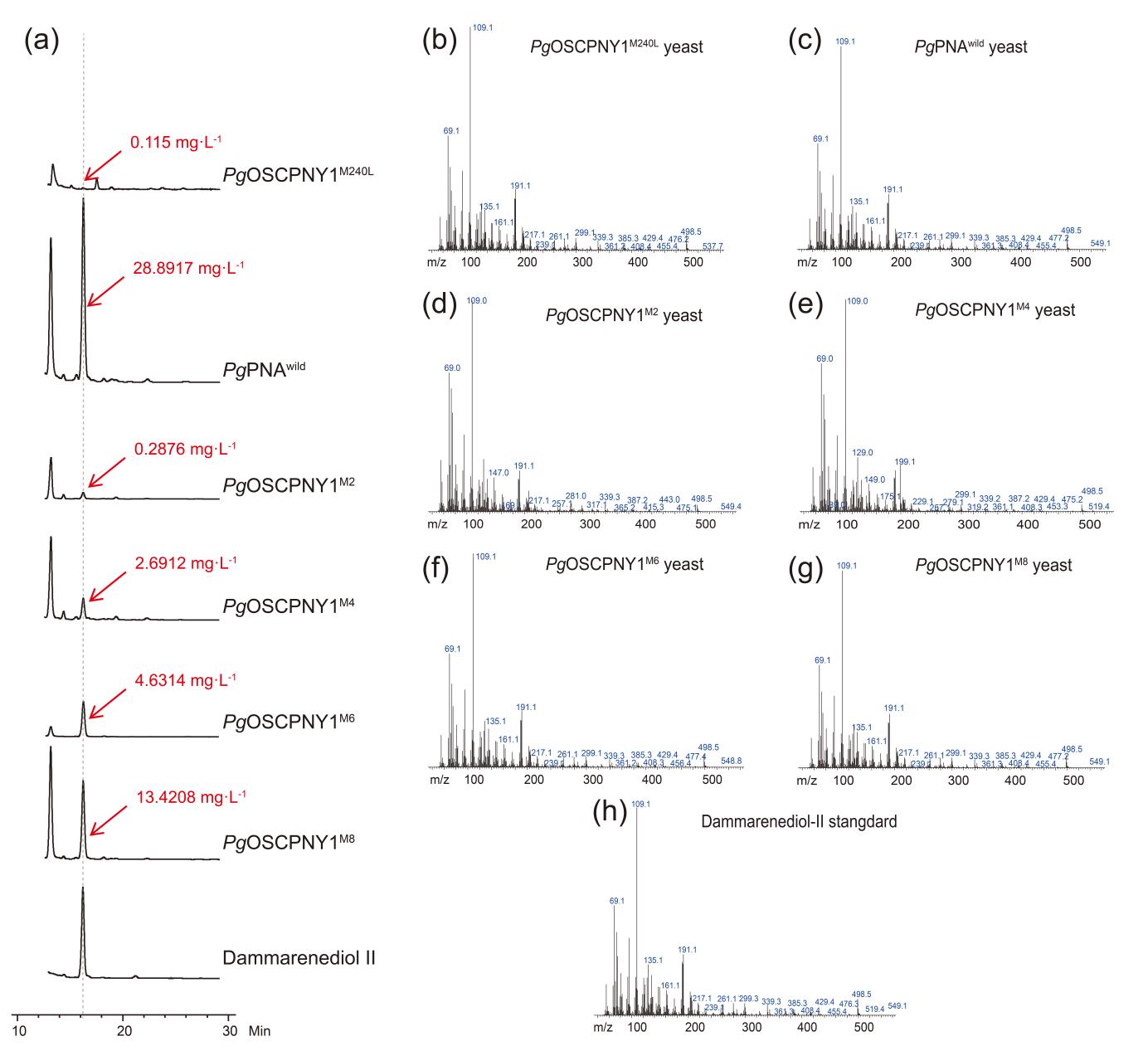
**Figure S4** Dammarenediol-II content in mutant *Pg*OSCPNY1 and wild-type *Pg*PNA.

(a) Detection of dammarenediol-II content using HPLC. (b-h) GC-MS mass spectrum analysis of dammarenediol-II (trimethylsilane derivatives).


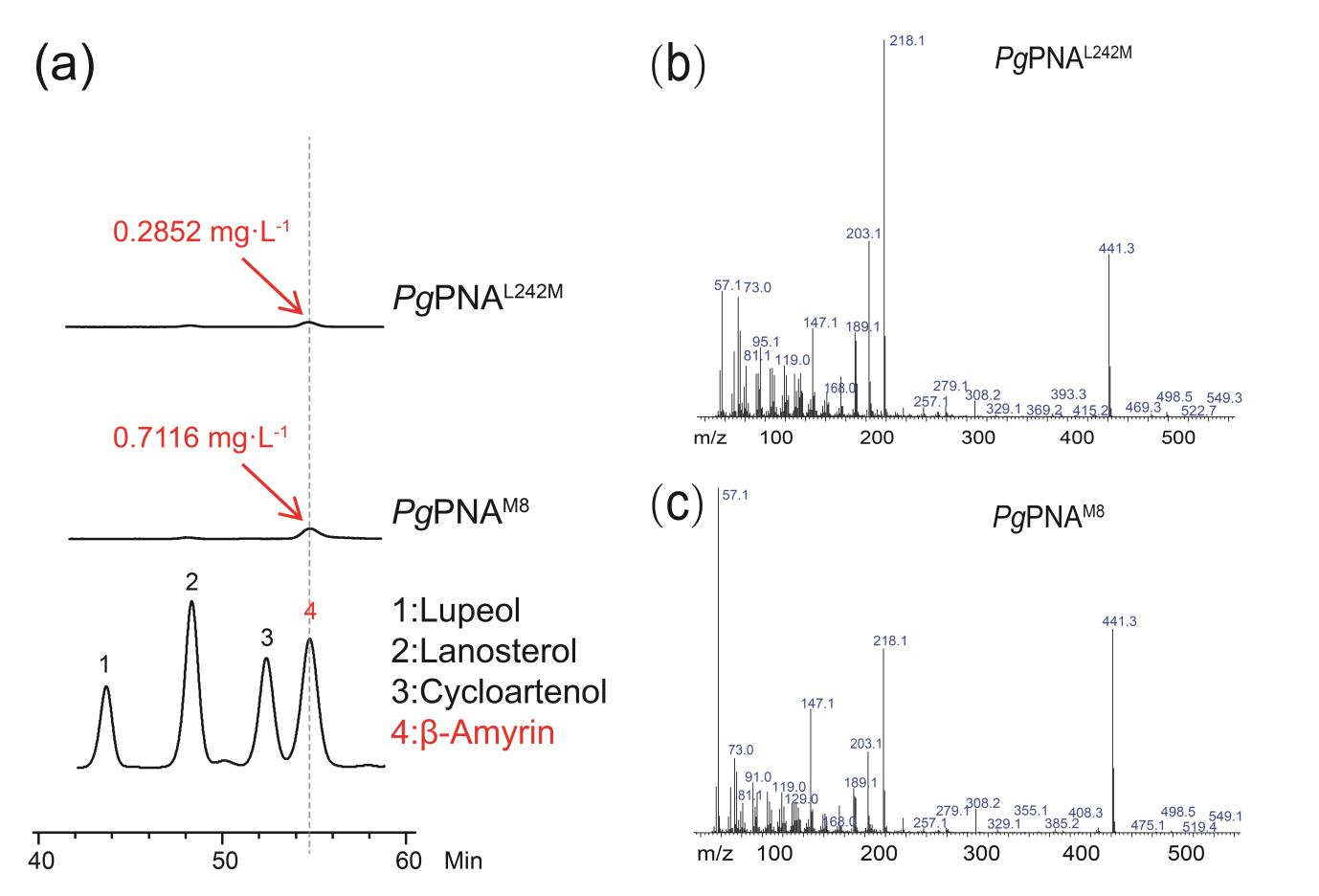
**Figure S5** *β*-amyrin content in mutant-type *Pg*PNA.

(a) Detection of *β*-amyrin content using HPLC. (b-c) GC-MS mass spectrum analysis of *β*-amyrin (trimethylsilane derivatives).


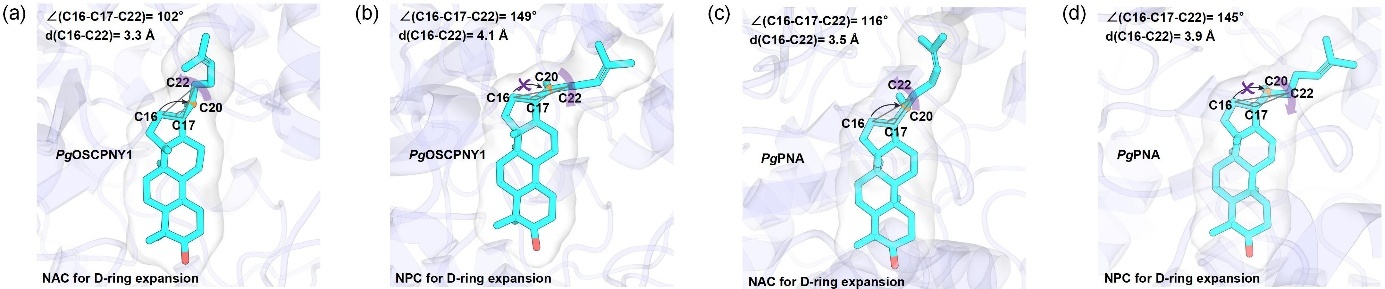


**Figure S6** Zoom of the active site showing, in *Pg*OSCPNY1 and *Pg*PNA, the NAC facilitating and the NPC inhibiting D-ring expansion.


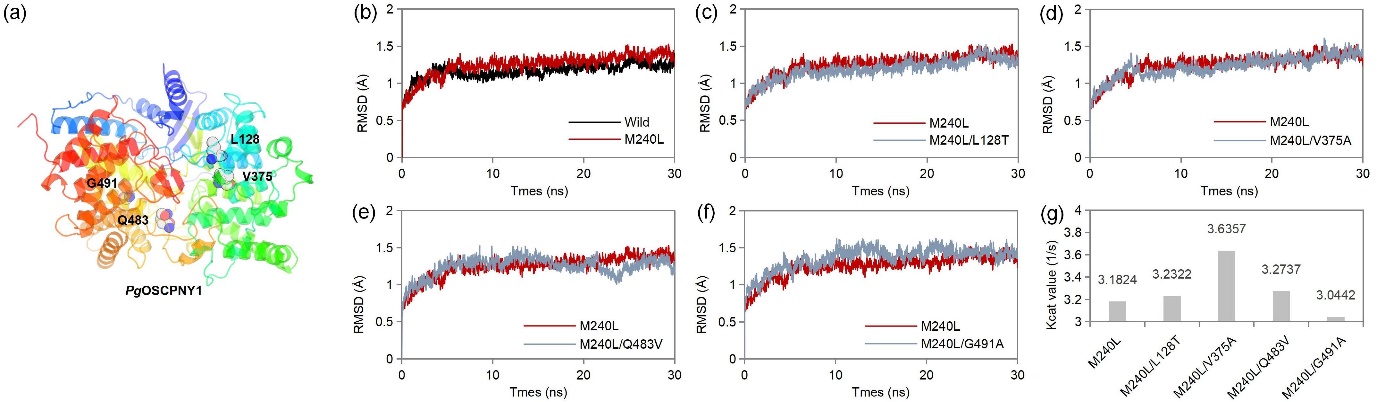


**Figure S7** The effects of L128T, V375A, Q483V, and G491A mutations on the stability and catalytic efficiency of the novel enzyme *Pg*OSCPNY1^M240L^.

(a) Overview of L128, V375, Q483 and G491 in *Pg*OSCPNY1. (b-f) RMSD trajectories of wild-type and mutant-type *Pg*OSCPNY1. (g) The Kcat values for mutant-type *Pg*OSCPNY1 predicted by DLKcat.


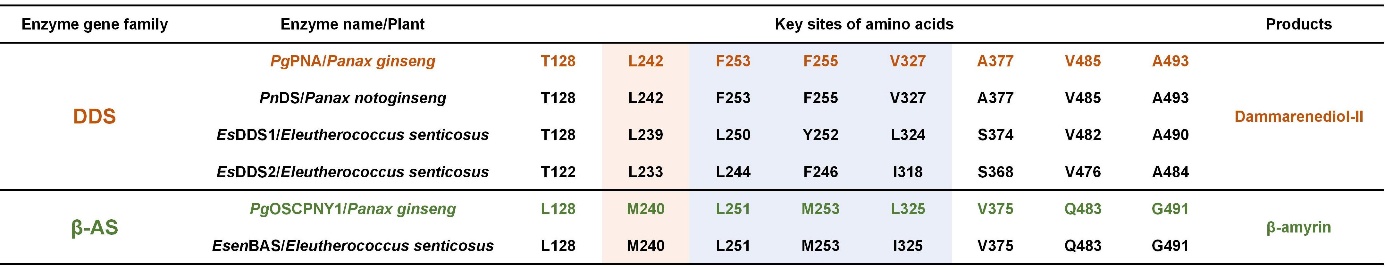


**Figure S8** Sequence alignments between DDSs and β-ASs from Araliaceae, including Panax ginseng, Panax notoginseng, and Eleutherococcus senticosus.


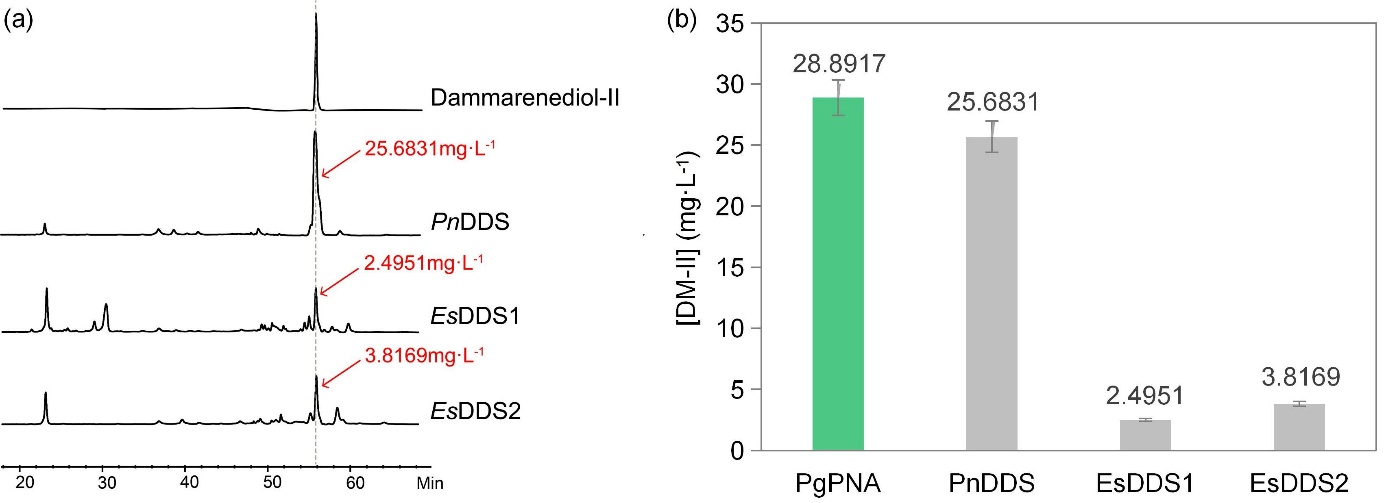


**Figure S9** Dammarenediol-II content in Araliaceae DDSs.

(a) Detection of dammarenediol-II content using HPLC. (b) Comparison of Araliaceae DDSs activities. Error-bars represent standard deviation SD (n=3 independent samples).

**Table S1** Primers used for cloning.

| Enzyme | Primer cloning | Primer sequence (5’→3’) |
| --- | --- | --- |
| *Pg*PNA | PY-*Pg*PNAF | cttggtaccgagctcggatccATGTGGAAGCAGAAGGGTG |
|  | PY-*Pg*PNAR | gccgccagtgtgatggatatctgcagaattcTTAAATTTTGAGCTG |
| *Pn*DS | PY-*Pn*DSF | cttggtaccgagctcggatccATGTGGAAGCAGAAGG |
|  | PY-*Pn*DSR | gccgccagtgtgatggatatctgcagaattcTTAAATTTTGAGCTGC |
| *Aa*OSC2 | PY-*Aa*OSC2F | cttggtaccgagctcggatccATGTGGAAGTTGAAGGTAGC |
|  | PY-*Aa*OSC2R | cggccgccagtgtgatggatatctgcagaattcTTATTTAGTTTTGAC |
| *Ca*DDS-like | PY-*Ca*DDS-likeF | cttggtaccgagctcggatccATGTGGAAGCTAAAGATTGCTG |
|  | PY*-Ca*DDS-likeR | gccgccagtgtgatggatatctgcagaattcCTACAGACATTGAG |
| *Ca*OSCCAS | PY-*Ca*OSCCASF | cttggtaccgagctcggatccATGTGGAAGCTGAAGATAGC |
|  | PY-*Ca*OSCCASR | gccagtgtgatggatatctgcagaattcTCAATTGGAGAGCCACAAGC |
| *Cc*DDS-like | PY-*Cc*DDS-likeF | cttggtaccgagctcggatccATGTGGAAGCTGAAAATTGGTG |
|  | PY-*Cc*DDS-likeR | cgccagtgtgatggatatctgcagaattcTTAGATGGCTTCTTGTTTCGG |
| *Cp*DDS1 | PY-*Cp*DDS1F | cttggtaccgagctcggatccATGTGTATATCCTTCAGGGAG |
|  | PY-*Cp*DDS1R | cgccagtgtgatggatatctgcagaattcTCAATCCTGCTGAAGATGG |
| *Cp*DDS2 | PY-*Cp*DDS2F | cttggtaccgagctcggatccATGTGGAAACTGAAGATAGC |
|  | PY-*Cp*DDS2R | cgccagtgtgatggatatctgcagaattcTTAGAACTTTATGGTGGG |
| *Cp*DDS3 | PY-*Cp*DDS3F | cttggtaccgagctcggatccATGTGGAAGCTAAAGATAGC |
|  | PY-*Cp*DDS3R | cgccagtgtgatggatatctgcagaattcTTACAACTTTTGGCTCAGC |
| *Cr*AS | PY-*Cr*ASF | cttggtaccgagctcggatccATGTGGAAGCTAAAGATAGCAG |
|  | PY-*Cr*ASR | gccagtgtgatggatatctgcagaattcCTACAAAGCTTTGGTTGGC |
| *Eg*DDS-like | PY-*Eg*DDS-likeF | gtaccgagctcggatccATGTGGAAGCTGAAG |
|  | PY-*Eg*DDS-likeR | cgccagtgtgatggatatctgcagaattcTTAAATATATATGTAAGTTAGGG |
| *Eg*DDS-like-X2 | PY-*Eg*DDS-like-X2F | cttggtaccgagctcggatccATGTGGAAGCTGAAGATAGC |
|  | PY-*Eg*DDS-like-X2R | cgccagtgtgatggatatctgcagaattcTTAAAGGGATTGAGATGGCC |
| *Es*DDS1 | PY-*Es*DDS1F | gtaccgagctcggatccATGTGGAAGCTGAAGATTG |
|  | PY-*Es*DDS1R | ccagtgtgatggatatctgcagaattcTCATGTTGAAGACTTTTCAAG |
| *Es*DDS2 | PY-*Es*DDS2F | cttggtaccgagctcggatccATGTGGAAGCTGAAGATTGC |
|  | PY-*Es*DDS2R | agtgtgatggatatctgcagaattcTCACATGTCGGGCTTAGACTTC |
| *Ha*DDS | PY-*Ha*DDSF | cttggtaccgagctcggatccATGTGGAAGTTGAAGATAGC |
|  | PY-*Ha*DDSR | ccgccagtgtgatggatatctgcagaattcTTAATTGATCCAAACTCG |
| *Ls*DDS-like | PY-*Ls*DDS-likeF | cttggtaccgagctcggatccATGTGGAAGTTAAAGATAGC |
|  | PY-*Ls*DDS-likeR | cgccagtgtgatggatatctgcagaattcTTAATTGACCCAAACACG |
| *Ob*OSC2 | PY-*Ob*OSC2F | cttggtaccgagctcggatccATGTGGAAGCTCAAGATTGC |
|  | PY-*Ob*OSC2R | cgccagtgtgatggatatctgcagaattcTTACAAGCATTGTGATGAC |
| *Oe*DDS-like | PY-*Oe*DDS-likeF | cttggtaccgagctcggatccATGTGGAAGCTTAAGATTGC |
|  | PY-*Oe*DDS-likeR | cgccagtgtgatggatatctgcagaattcTTACAGGCTTTGAGATGAC |
| *Ofβ*-AS | PY-*Ofβ*-ASF | cttggtaccgagctcggatccATGTGGAAGCTTAAGATTGC |
|  | PY-*Ofβ*-ASR | gccagtgtgatggatatctgcagaattcTTACAGGCTTTGAGATGGC |
| *Si*DDS-like | PY-*Si*DDS-likeF | cttggtaccgagctcggatccATGTGGAAGCTGAAGATTG |
|  | PY-*Si*DDS-likeR | ccagtgtgatggatatctgcagaattcTTAGAGGCATTGAGATGGCC |
| *Tc*OSC1 | PY-*Tc*OSC1F | cttggtaccgagctcggatccATGTGGAAGCTGAGAATAGG |
|  | PY-*Tc*OSC1R | gccgccagtgtgatggatatctgcagaattcTTAGGTTTCTTGTTTTGG |
| *Tk*OSC3 | PY-*Tk*OSC3F | cttggtaccgagctcggatccATGTGGGAGTTAAAGATAGC |
|  | PY-*Tk*OSC3R | ggccgccagtgtgatggatatctgcagaattcTTAATTTTGAATACCC |
| *Pg*OSCPNY1 | PY-*Pg*OSCPNY1F | cttggtaccgagctcggatccatgtggaagcttaagatagc |
|  | PY-*Pg*OSCPNY1R | cgccagtgtgatggatatctgcagaattccttaggtgcctaggg |
| *Et*AS | PY-*Et*ASF | cttggtaccgagctcggatccatgtggaagctgaagatagc |
|  | PY-*Et*ASR | ccgccagtgtgatggatatctgcagaattcttaaagagtagtggaaggc |
| *Gs*AS1 | PY-*Gs*AS1F | gtaccgagctcggatccatgtggaggctaaaaatcgg |
|  | PY-*Gs*AS1R | cagtgtgatggatatctgcagaattcctacggcacttgcttgc |
| *Gs*AS2 | PY-*Gs*AS2F | ggtaccgagctcggatccatgtggaggctgaagatc |
|  | PY-*Gs*AS2R | cagtgtgatggatatctgcagaattctcaagatggcaatggcactc |
| *Ob*asOSC1 | PY-*Ob*asOSC1F | cttggtaccgagctcggatccatgtggaggctgaagattg |
|  | PY-*Ob*asOSC1R | cgccagtgtgatggatatctgcagaattcttatgttgctcttgatggaag |
| *Tw*OSC2 | PY-*Tw*OSC2F | cttggtaccgagctcggatccatgtggaggctaaagattg |
|  | PY-*Tw*OSC2R | ccgccagtgtgatggatatctgcagaattcctagactttggaagataatg |
|  |  |  |
| *Pg*OSCPNY1^M240A^ | PY-*Pg*OSCPNY1M240A-F | AgctCCCCCAGAATTCTGGATTCTCCCTTCTT |
|  | PY-*Pg*OSCPNY1M240A-R | AGAATTCTGGGGGagcTGGGTTGCTTCCTATCCATTCA |
| *Pg*OSCPNY1^L251A^ | PY-*Pg*OSCPNY1L251A-F | TTCTTTCgctCCTATGCACCCAGCTAAAATGTG |
|  | PY-*Pg*OSCPNY1L251A-R | GCATAGGagcGAAAGAAGGGAGAATCCAGAATTCT |
| *Pg*OSCPNY1^M253A^ | PY-*Pg*OSCPNY1M253A-F | TTTCCTTCCTgctCACCCAGCTAAAATGTGGTGTTAT |
|  | PY-*Pg*OSCPNY1M253A-R | GGTGagcAGGAAGGAAAGAAGGGAGAATCCAG |
| *Pg*OSCPNY1^L325A^ | PY-*Pg*OSCPNY1L325A-F | AGACCTGgctTGGGATAGTCTCTATGTATTAACTGAACC |
|  | PY-*Pg*OSCPNY1L325A-R | TATCCCAagcCAGGTCTTGTATTAAAGGATGAGGATA |
| *Pg*OSCPNY1^L128A^ | PY-*Pg*OSCPNY1L128A-F | TTTTTCgctCCGCCCTTGGTGATGTGTGTATA |
|  | PY-*Pg*OSCPNY1L128A-R | AAGGGCGGagcGAAAAAGAGAGGTCCGGAATTCT |
| *Pg*OSCPNY1^V375A^ | PY-*Pg*OSCPNY1V375A-F | GTGGAAAAGgctTTGTGTATGCTTGTTTGTTGGGTT |
|  | PY-*Pg*OSCPNY1V375A-R | CACAAagcCTTTTCCACACATCCAATGGTAAT |
| *Pg*OSCPNY1^Q483A^ | PY-*Pg*OSCPNY1Q483A-F | ACGGATGGgctGTTTCTGATTGCACTGCAGAAGG |
|  | PY-*Pg*OSCPNY1Q483A-R | AGAAACagcCCATCCGTGATCTTGATCTGAAA |
| *Pg*OSCPNY1^M240L^ | PY-*Pg*OSCPNY1M240L-F | CCAttgCCCCCAGAATTCTGGATTCTCCCTTC |
|  | PY-*Pg*OSCPNY1M240L-R | AATTCTGGGGGcaaTGGGTTGCTTCCTATCCATTCA |
| *Pg*OSCPNY1^L251F^ | PY-*Pg*OSCPNY1L251F-F | CTTTCttcCCTATGCACCCAGCTAAAATGTGG |
|  | PY-*Pg*OSCPNY1L251F-R | GTGCATAGGgaaGAAAGAAGGGAGAATCCAGAATTCT |
| *Pg*OSCPNY1^M253F^ | PY-*Pg*OSCPNY1M253F-F | TCCTTCCTttcCACCCAGCTAAAATGTGGTGTTAT |
|  | PY-*Pg*OSCPNY1M253F-R | TGGGTGgaaAGGAAGGAAAGAAGGGAGAATCC |
| *Pg*OSCPNY1^L325V^ | PY-*Pg*OSCPNY1L325V-F | AGACCTGgttTGGGATAGTCTCTATGTATTAACTGAACC |
|  | PY-*Pg*OSCPNY1L325V-R | TATCCCAaacCAGGTCTTGTATTAAAGGATGAGGATA |
| *Pg*OSCPNY1^L128T^ | PY-*Pg*OSCPNY1L128T-F | CTTTTTCactCCGCCCTTGGTGATGTGTGTAT |
|  | PY-*Pg*OSCPNY1L128T-R | AGGGCGGagtGAAAAAGAGAGGTCCGGAATTCT |
| *Pg*OSCPNY1^V375S^ | PY-*Pg*OSCPNY1V375S-F | GTGGAAAAGtctTTGTGTATGCTTGTTTGTTGGGTT |
|  | PY-*Pg*OSCPNY1V375S-R | CACAAagaCTTTTCCACACATCCAATGGTAAT |
| *Pg*OSCPNY1^Q483V^ | PY-*Pg*OSCPNY1Q483V-F | CGGATGGgttGTTTCTGATTGCACTGCAGAAGG |
|  | PY-*Pg*OSCPNY1Q483V-R | CAGAAACaacCCATCCGTGATCTTGATCTGAAA |
| PgOSCPNY1^G491A^ | PY-*Pg*OSCPNY1G491A-F | CTGCAGAAgctCTAAAGTGTTGCCTTATTTTCTCAACG |
|  | PY-*Pg*OSCPNY1G491A-R | CTTTAGagcTTCTGCAGTGCAATCAGAAACTTG |
| *Pg*PNA^L242M^ | PY-*Pg*PNAL242M-F | GatgCCCCCAGAATTCTGGCTTTTCCCTTCAA |
|  | PY-*Pg*PNAL242M-R | AGAATTCTGGGGGcatCGGGTTGCACCCTTCCCA |
| *Pg*PNA^F253L^ | PY-*Pg*PNAF253L-F | CCCTTCAAGTcttCCTTTTCATCCAGCAAAAATGTG |
|  | PY-*Pg*PNAF253L-R | AAGGaagACTTGAAGGGAAAAGCCAGAATTCT |
| *Pg*PNA^F255M^ | PY-*Pg*PNAF255M-F | TTTCCTatgCATCCAGCAAAAATGTGGATCTAC |
|  | PY-*Pg*PNAF255M-R | GCTGGATGcatAGGAAAACTTGAAGGGAAAAGCC |
| *Pg*PNA^V327L^ | PY-*Pg*PNAV327L-F | AAGACCTGctcTGGGATGGTCTTCACTACTTTAGTGA |
|  | PY-*Pg*PNAV327L-R | ATCCCAgagCAGGTCTTGTACAAGGGTATGAGGG |
| *Pg*PNA^T128L^ | PY-*Pg*PNAT128L-F | ACTTTATcttCCTCCCCTTATCATTGCCCTAT |
|  | PY-*Pg*PNAT128L-R | GGGGAGGaagATAAAGTAAAGAGCCTGCATTTTCAG |
| *Pg*PNA^A377V^ | PY-*Pg*PNAA377V-F | GGGGAAAAAgttTTACAAATAATGAGTTGGTGGGCA |
|  | PY-*Pg*PNAA377V-R | TGTAAaacTTTTTCCCCATTTCCTGTGGTTAT |
| *Pg*PNA^V485Q^ | PY-*Pg*PNAV485Q-F | ATGGTTGCcaaGTCTCGGACTGCACAGCTGAA |
|  | PY-*Pg*PNAV485Q-R | CGAGACttgGCAACCATGATCTTGATCAGAGAA |
| *Pg*PNA^A493G^ | PY-*Pg*PNAA493G-F | CAGCTGAAggaCTAAAGTGCCTACTGTTACTTTCACAAA |
|  | PY-*Pg*PNAA493G-R | CTTTAGtccTTCAGCTGTGCAGTCCGAGACAA |

**Table S2** YPD medium formula.

| Reagent | Volume |
| --- | --- |
| Yeast extract | 10g/L |
| Peptone | 20g/L |
| Glucose | 2% |
| pH | 7.0 |
| Agar powder（Solid medium） | 20g/L |

**Table S3** SC-Ura medium formula.

| Reagent | Volume |
| --- | --- |
| YNB | 0.67% (6.7g/L) |
| Threonine | 0.1 g/L |
| Lysine | 0.1 g/L |
| Cysteine | 0.1 g/L |
| Tryptophan | 0.1 g/L |
| Arginine | 0.1 g/L |
| Leucine | 0.1 g/L |
| Valine | 0.05 g/L |
| Tyrosine | 0.05 g/L |
| Proline | 0.05 g/L |
| Serine | 0.05 g/L |
| Methionine | 0.05 g/L |
| Isoleucine | 0.05 g/L |
| Aspartic acid | 0.05 g/L |
| Phenylalanine | 0.05 g/L |
| Histidine | 0.05 g/L |
| AdenIne | 0.1 g/L |
| Liquid medium | pH 5.6 |
| Solid medium | pH 6.5 |
| Agar powder（Solid medium） | 20 g/L |

**Table S4** HPLC procedures for products^a^.

| Time（min） | A: Pure water | B: Acetonitrile | The velocity（mL/min） | Max pressure（bar） |
| --- | --- | --- | --- | --- |
| 0 | 30.0 | 70.0 | 0.8 | 400.0 |
| 25 | 10.0 | 80.0 | 0.8 |  |
| 33 | 8.0 | 92.0 | 0.8 |  |
| 37 | 0.0 | 100.0 | 0.8 |  |
| 70 | 0.0 | 100.0 | 0.8 |  |

^a^HPLC detection of OSCs (*Pg*PNA, *Pn*DS, *Aa*OSC2, *Ca*DDS-like, *Ca*OSCCAS, *Cc*DDS-like, *Cr*AS, *Eg*DDS-like, *Eg*DDS-like-X1, *Es*DDS1, *Es*DDS2, *Ha*DDS, *Ls*DDS-like, *Ob*OSC2, *Oe*DDS-like, *Of*β-As, *Si*DDS-like, *Tc*OSC1, *Tk*OSC3) products.

**Table S5** HPLC procedures for products^b^.

| Time（min） | A: Pure water | B: Acetonitrile | The velocity（mL/min） | Max pressure（bar） |
| --- | --- | --- | --- | --- |
| 0 | 75.0 | 25.0 | 0.8 | 400.0 |
| 25 | 30.0 | 70.0 | 0.8 |  |
| 33 | 0.0 | 100.0 | 0.8 |  |
| 60 | 0.0 | 100.0 | 0.8 |  |

**^b^**HPLC detection of OSCs (*Cp*DDS1, *Cp*DDS2, *Cp*DDS3) products.

**Table S6** HPLC procedures for products^c^.

| Time（min） | A: Pure water | B: Acetonitrile | The velocity（mL/min） | Max pressure（bar） |
| --- | --- | --- | --- | --- |
| 0 | 5.0 | 95.0 | 0.8 | 400.0 |
| 5 | 5.0 | 95.0 | 0.8 |  |
| 10 | 0 | 100.0 | 0.8 |  |
| 60 | 0 | 100.0 | 0.8 |  |

**^c^**HPLC detection of *Pg*PNA, *Pg*OSCPNY1 and their mutant products.

**Table S7** LC-MS procedures for products^d^.

| Time（min） | A: Pure water | B: Acetonitrile | The velocity（mL/min） | Max pressure（bar） |
| --- | --- | --- | --- | --- |
| 0 | 5.0 | 95.0 | 1.0 | 400.0 |
| 2 | 5.0 | 95.0 | 1.0 |  |
| 10 | 0 | 100.0 | 1.0 |  |
| 30 | 0 | 100.0 | 1.0 |  |

**^d^**LC-MS detection of 22 DDS products.

**Data S1** Gene sequences of enzymes.

**>*Pg*PNA (AB265170.1)/*Panax ginseng* (2310bp)**

atgtggaagcagaagggtgcccaaggaaatgatccatatttgtatagcactaacaactttgttggcagacaatattgggagtttcagcccgatgctggtactccagaagagagggaagaggttgaaaaagcacgcaaggattatgtaaacaataagaagctacatggaattcatccatgcagtgatatgctgatgcgcaggcagcttattaaagaaagtggaatcgatctcctaagcataccgccgttgagattagatgaaaacgaacaagtgaactacgatgcagttacaaccgctgtgaagaaagctcttcgattgaaccgggcaattcaagcacacgatggtcactggccagctgaaaatgcaggctctttactttatacacctccccttatcattgccctatatatcagcggaacgattgacactattctgacaaaacaacacaagaaggaactgattcgcttcgtttacaaccatcaaaatgaggatggtggatggggatcctatattgaggggcacagcacgatgattgggtcagtacttagctacgtgatgttacgtttgctaggagaaggattagctgaatctgatgatggaaatggtgcagttgagagaggccggaagtggatacttgatcatggaggtgcagccggcataccctcttggggaaagacttatctagcggtgcttggagtatatgagtgggaagggtgcaacccgctgcccccagaattctggcttttcccttcaagttttccttttcatccagcaaaaatgtggatctactgccggtgcacttacatgccaatgtcgtatttgtatgggaagagatatcatggaccaataaccgatcttgttttatctttgaggcaagaaatttacaacattccttatgagcagataaagtggaatcaacagcgccataactgttgcaaggaggatctctactaccctcatacccttgtacaagacctggtttgggatggtcttcactactttagtgaaccattcctcaaacgttggcccttcaacaaactgcgaaaaagaggtctaaaaagagttgttgaactaatgcgctatggtgccaccgagaccagattcataaccacaggaaatggggaaaaagctttacaaataatgagttggtgggcagaagatcccaatggtgatgagtttaaacatcaccttgctagaattcctgatttcttatggattgctgaggatggaatgacagtacagagttttggtagtcaactatgggactgtattcttgctactcaagcaattatcgccaccaatatggttgaagaatacggagattctcttaagaaggcgcatttcttcatcaaagaatcgcagataaaagaaaatccaagaggagacttcctaaaaatgtgtcgacagtttaccaaaggtgcgtggactttctctgatcaagatcatggttgcgttgtctcggactgcacagctgaagcgctaaagtgcctactgttactttcacaaatgccacaggatattgtcggagaaaaacctgaggttgagcgattatatgaggctgtgaatgttcttctctatttgcagagtcgtgtaagtggtggtttcgcagtttgggagcctccagttccaaaaccatatttggagatgttgaatccttcagaaatttttgcagacattgttgttgagagagagcacattgaatgcactgcatctgtaatcaaaggtctgatggcatttaaatgcttgcatcctgggcatcgtcagaaagagatagaggattctgtggcgaaagccatccgttatcttgaaagaaaccaaatgcctgatggttcatggtatggcttttggggaatttgtttcctctatgggacattttttaccctatctgggtttgcttctgctgggaggacttatgacaacagtgaagcagttcgtaagggtgttaaatttttcctttcaacacaaaatgaagaaggtggttggggggagagtcttgaatcatgcccaagcgaaaaatttacaccactcaagggaaacagaacaaatctagtacaaacatcatgggctatgctaggtcttatgtttggtggacaggccgagagagatccgacacctctgcatagagcagcaaagttgttgatcaatgcgcaaatggataatggagatttccctcaacaggaaattactggagtatactgtaaaaatagtatgttacattatgcggagtacagaaatatatttcctctttgggcactcggagaatatcggaaacgtgtttggttgcctaagcaccagcagctcaaaatttaa

**>*Pn*DS (KC422652.1)/*Panax notoginseng* (2310bp)**

atgtggaagcagaagggtgcccaaggaaatgatccatatttgtatagcactaacaactttgttggcagacaatattgggagtttcagcccgatgctggtactccagaagagagggaagaggttgaaaaagcacgcaaggattatgtaaacaataagaagctacatggaattcatccatgcagtgatatgctgatgcgcaggcagcttattaaagaaagtggaatcgatctcctaagcataccgccgttgagattagatgaaaacgaacaagtgaactacgatgcagttacaaccgctgtgaagaaagctcttcgattgaaccgggcaattcaagcacacgatggtcactggccagctgaaaatgcaggctctttactttatacacctccccttatcattgccctatatatcagcggaacgattgacactattctgacaaaacaacacaagaaggaactgattcgcttcgtttacaaccatcaaaatgaggatggtggatggggatcctatattgaggggcacagcacgatgattgggtcagtacttagctacgtgatgttacgtttgctaggagaaggattagctgaatctgatgatggaaatggtgcagttgagagaggccggaagtggatacttgatcatggaggtgcagccggcataccctcttggggaaagacttatctagcggtgcttggagtatatgagtgggaagggtgcaacccgctgcccccagaattctggcttttcccttcaagttttccttttcatccagcaaaaatgtggatctactgccggtgcacttacatgccaatgtcgtatttgtatgggaagagatatcatggaccaataaccgatcttgttttatctttgaggcaagaaatttacaacattccttatgagcagataaagtggaatcaacagcgccataactgttgcaaggaggatctctactaccctcatacccttgtacaagacctggtttgggatggtcttcactactttagtgaaccattcctcaaacgttggcccttcaacaaactgcgaaaaagaggtctaaaaagagttgttgaactaatgcgctatggtgccaccgagaccagattcataaccacaggaaatggggaaaaagctttacaaataatgagttggtgggcagaagatcccaatggtgatgagtttaaacatcaccttgctagaattcctgatttcttatggattgctgaggatggaatgacagtacagagttttggtagtcaactatgggactgtattcttgctactcaagcaattatcgccaccaatatggttgaagaatacggagattctcttaagaaggcgcatttcttcatcaaagaatcgcagataaaagaaaatccaagaggagacttcctaaaaatgtgtcgacagtttaccaaaggtgcgtggactttctctgatcaagatcatggttgcgttgtctcggactgcacagctgaagcgctaaagtgcctactgttactttcacaaatgccacaggatattgtcggagaaaaacctgaggttgagcgattatatgaggctgtgaatgttcttctctatttgcagagtcgtgtaagtggtggtttcgcagtttgggagcctccagttccaaaaccatatttggagatgttgaatccttcagaaatttttgcagacattgttgttgagagagagcacattgaatgcactgcatctgtaatcaaaggtctgatggcatttaaatgcttgcatcctgggcatcgtcagaaagagatagaggattctgtggcgaaagccatccgttatcttgaaagaaaccaaatgcctgatggttcatggtatggcttttggggaatttgtttcctctatgggacattttttaccctatctgggtttgcttctgctgggaggacttatgacaacagtgaagcagttcgtaagggtgttaaatttttcctttcaacacaaaatgaagaaggtggttggggggagagtcttgaatcatgcccaagcgaaaaatttacaccactcaagggaaacagaacaaatctagtacaaacatcatgggctatgctaggtcttatgtttggtggacaggccgagagagatccgacacctctgcatagagcagcaaagttgttgatcaatgcgcaaatggataatggagatttccctcaacaggaaattactggagtatactgtaaaaatagtatgttacattatgcggagtacagaaatatatttcctctttgggcactcggagaatatcggaaacgtgtttggttgcctaagcaccagcagctcaaaatttaa

**>*Aa*OSC2 (KF309252.1)/*Artemisia annua* (2283bp)**

atgtggaagttgaaggtagcagaaggaaatgatccttacttgttttcaaccaacaattttgtcggtaggcaaatttgggaatttgatcccagtgcaggaagtccggtcgagcgtcaagaagtagaagatgctcgtcaacaatttaaaaacaatcggagggaaggtgttcatccatgcggtgatttgcttatgcggatccagttaatcaaagaaaatggaattgatgtaatgagcataccaccagtgcgattgggagagaatgaggatgtgaattatgacgcagtgacaacgacagtgaagaaggcacttcgattgaaccgtgccatccaagcaaaagatggtcactggcctgccgaaaatgctggccctatgtttttcactcctccccttcttattgctatgtacatcagtggaaccatcaacacacatttaaccaaagagcacaggacggaaatgataagatatatctacaaccatcaaaatgaagatggagggtggggattttatattgagggacacagcaccatgatcggatctgcattgagctatgtagcattgagattgctaggagaaggacctgacgatggaaatggagcagtggaccgcgccaggaagtggatactagaccatggtggtgcagcctccattccatcttggggaaaaacttatctatcggtgcttggggtatatgaatgggaagggtgcaatccacttccaccagaattttggctttttcctgaagctttaccatttcatccagcaaaaatgtggtgctattgccggacaacttatatgccaatgtcatacttgtacgggagaaaataccatggtccaatcactgatcttgttctgcaacttcgacaagaaattcatcccatcccatatcacaagatcaactggaataaacagcgccataactgttgcaaggaagatctctactaccctcattcaacagtccaagatttgttgtgggatggtcttcattacttgagtgaaccaatccttaagtactggcctttcaccaaactgagagaaagaggtctgaaaagagcagttgaactaatgcgttatggtgctgaagagagcagatatatgaccattggatgcgttgaaaagagcttgcaaatgatgtgttggtgggcggagaacccaaatggggatgagttcaagcatcaccttgctagagtgcctgattacttgtggctggctgaagatgggatgacaatgcatagttttgggagccaactatgggattgcgtactcgcaactcaggcaataatcgctagtgatatggtcgaagaatttggggattcacttaaaaaggcccatttttatatcaaagaatctcaaattaaacaaaatccttctggtgactttagtaagatgtgccggcaatttactaaaggggcgtggactttctctgaccaagatcaaggttgggttgtctcagactgtacagctgaagcattgaagtgtcttctgttactatctcaaatgccagaggaaatttctggaaaaaaagcagataatgagcggttatatgaggctgttaacgtccttttatacttacagagtcctataagtggaggttttgcaatttgggagccaccagtcccgcaaccatatttacagatgttgaatccttcagaaattttcgcagacattgtagttgagaaagagcatgttgagtgcacatcatcgattatcaaagccctcttagccttcaaagatttacacccaggacacagggagaaagaaatagaaatttctgtggcgaaagcagtttgctttttggagggaaagcaatgtcatgacggttcatggtatggttattggggaatatgcttcctatatggcacattctttactttagcaggcctagtttctgctggaaaaacgtatgacaatagcgaagcagttcgtaaagctgtcaactttttcctttcgacgcaaaatgaagagggtggatggggagagagtatcaagtcttgccctagtgaagtatacacaccgttggatggaaatcggacaaacctagttcaaacatcatgggctatgcttggtcttatgttcggtggacaggccgaaagggatccaacacccttgcataaagcagcaaaaatattaattaacgcacagatggatgacggggattttcctcaacaggagattaccggagtctacatgaagaactgcatgctacattatgcagaatacaggaacattttcccgctttgggcacttggagaatatcgcaaacgagtttgggtcaaaactaaataa

**>*Ca*DDS-like (XM_027232213.1)/*Coffea arabica* (2286bp)**

atgtggaagctaaagattgctgaaggccacggaccttacctgtacagcaccaacaattttgccggcaggcaaatttgggagtatgatcccaatgctggaacgcctgaggaaagagaagcatttcaaaaagctcgagaggaattcacaaaaaataggaagaagggtgttcatccatgtggtgatttgttcatgagaatgcagcttataaaagaaagtggaattgatctgttgagcatcccaccaattagattgggagaaaacgaagaagtaacctatgaaaatactactattgctgttaagaaagcacttaggctaaaccgtgcaatccaggcaagcgatggccattggccggctgagaatgccgggccaatgtttttcacgccgccactgctcattgcattgtacatcagtggagcaattaacactatcctgacaaaagaacacaaaaaagagatgattcgttacatctacaaccatcaaaatgatgatggtggttggggattctacattgagggtcacagcaccatgattggatcagccctcagctatgtagctttacgaattctcggcgaaggacccgatgatggaaatggtgcagttgctagagctcgcaaatggatccttgatcatggcggtgcaactggaattccctcctggggaaagacttatctctcggtacttggagtgtacgactgggatggctgcaacccagtgcctccagagttttggcttttccctgaatttttcccttatcatccagcaaagatgtggtgttactgccggacaacctacatgcccatgtcctacttgtatggcaaaaaatatcacgggccgcttactgatcttgttatgtcactcagacaggaaattcatgtaaaaccatatgatcaaattgactggaataaagcacgccatgactgttgcaaggaggacttgtactaccctcattcccgcatccaagatcttctctgggatacactcaattattgcacagagccaattatgagacgttggccattgaacaaaatcagagaaagagctatggacaaagcaattaaatacatgcggtatggagctgaagagagcagatacattaccattggatgtgttgaaaagagtttacaaatgatgtgttggtgggcgcacgatccaaattgcgatgagttcaagtatcacctggctagagttcccgattacctatggcttgctgaagatgggatgaaaatgcagagctttggaagtcaggtctgggattgtgcacttgctactcaggctgttatggcaactgggatggtagatgaatatggtgactgtctgacgaaagcacatttccacattaaagaatcccaggttaaagaaaatcccaaaggtgacttcaaaagcatgtaccgtcacttcaccaaaggttcttggactttctctgatcaagatcaagggtgggttgtctcagattgtactgctgaatcactcaagtgtctcttgatgctttcgcaaatgccaacagaaatttctggcaaaaaagccgatgtggagcgcctgtatgatgcagtgaatgttcttctctatctacagagtcctgagagtggtggcttttctatctgggaacctccagttccacaaccttatctgcaagttttgaatccttcagagctttttgcagacattgtagttgagcaagagcatgttgagaacactgcatccattatacaagctcttctattattcaagaggctccatccaggacacagggaaaaagagatagaaatagctgtggcaaaagctgtttcatttcttgaaggaaaacaatggcctgatggttcatggtatggctactggggtatttgcttcctgtatggaacaatgtttgtgttagctgggctagttgttgctggaaaaacttacagcaattctgaagcagttcgtaaagctgtccaattttatctatcaactcagaatgaagagggtggttggggagaatcccttgaatcttgccccagcatgaaatacacacctttggaaggcaatagaacaaatttagttcaaacagcatgggctatgcttggtcttatgcatggtggacaggctgagagagacccaacacctttgcacaaagcagccaaattattgatgaatgcacagatggatgatggagattttcctcaacaggaaattacaggggtctatatgaagaactgtatgcttcactatgcccaatataggaatatcttcccattgtgggcactttcagaatatcgtaaacgtgtctggccttctcaatgtctgtag

**>*Ca*OSCCAS (AY520818.1)/*Centella asiatica* (2283bp)**

atgtggaagctgaagatagcagagggtaatggagcatacttgtacagcaccaacaactttgtggggagacagatatgggagtatgatcctgatgctggaactcctgaagagcgactagaggtcgagaaacttcgagaaacttacaaatataatctcatcaacaatgggattcacccttgtggtgatatgctcatgaggttgcagctgataaaggagagtgggctggatcttttgagcataccgccggtgagacttggagaacaagaagaagtgaattatcaagtagtgacgacggctgttaagaaagctctgcggttaaaccgcgcaatccaagctcacgacggtcactggccagctgaaaatgctggacctatgttttttacaccacccctcatcatagcgctatacatcagcggagcaattgacactcatctaacaatacaacacaagaaggagatgattcgttttatttacctccaccaaaacaaagatggaggatggggattctatatagagggacatagcacgatgatagggtcagcacttagctacgtggcgttacgtttgctgggagaagggcctgatgacggcgatggtgcagtggagagagcaagaaaatggatccttgaccatggtggtgctgcttctataccctcctggggtaagacttatcttgcggttcttggggtatacgagtgggaagggtgtaaccccctgcccccagaattttggcttttccctgaagctttaccttatcatccagcaaaaatgtggtgttactgtcgcacaacatacatgccgatgtcgtatttgtatgggaagaaatatcatggtccaattacggatcttgttatatctttaagaaaagaaatacaccccattccttatgagaagataaattggaacaaacagcgccataactgtaacaaggaggatctttactaccctcatagctttatacaggatttgctatgggatggtcttcactattttactgaacctatcattaaaatgtggcccttcaataagttgcgaaagaaagggatgaaaagagccattgaacttatgcgctacggaggttatgagagcagattcattaccattggatgtgtatccaagagtctagatatgatgtgttggtgggcagagaacccgaatggtccagaattcaaacatcacttagctagagtacctgattacttgtggcttgcagaggatggaatgaagatgcagagttttggtagtcaattatgggactgtgttcttgctactcaagctgtcatgtctactggtatggttgatgaatatggggattgtcttaagaaagcacatttctatattaaagaatcacagtgcaagaaaaatccgtcaggagattatgcaagtatgtgccggtattttacaaaaggatcatggacattttctgatcaagatcagggttgggttgtctctgattgcacagctgaagcgctgaagtgtctattagcactttctcaaatgccagaggaaattgcaggggaaaaggcagatgttgagcgattatatgatgccgtaaacgtcctcctctacttgcaaagccctataagtgggggttttgccatttgggagccaccagttccaagaccatacttgcaggtgttgaatccttcggagatttttgccgacatcgttgtcgaaaaagagcatacggagtgcacagcatcaataatagcagctctggtagcattcaaacgtttgcatccgggtcatcggtcgaaagaaataagtgttgccatcgcaaaagctgtacattttcttgaaggaaaacaattggaagatggttcatggtatggctactggggaatatgctttttgtatggcacattttttgcgttagctgggttagcttctgtgggacagacttatgaaaacagtgaaaccgttcgtaaagctgttaagtttttcctttctacacaaaatgaagaaggtggttggggagaaagtcttgaatcatgtccgagcgagatattcacaccattagaaggaaacagaacaaatttagtacaaacatcatgggcaatgcttggtctcatgtttggtggacaggccacgagagatccaactccattgcatagagcagcaaagttattgattaatgcacaattgaataacggagatttccctcagcaggaaacaactggagtgtacatgaagaattgtatgttgcattatgccgagtatagaaatgtatttccgttatgggcacttggagagtaccgcaaacgcttgtggctctccaattga

**>*Cc*DDS-like (XM_025137525.1)/*Cynara cardunculus var.* (2304bp）**

atgtggaagctgaaaattggtgcgggaaatgacgacgaatatttgtacagcacaaacaattttgtgggacgtcaaacgtgggagttcgatcccgatgcaggcacgcaggaagagcgtgaccaagtcgaacgagctagagagcagttcttgatcaataagaaaaagcttgacataagttgttgcggggatttgctaatgagagctcagcttattaaggaaagcgggattgatctactgagcgaacctccaataagactcggagaagaagaggacgtgagctatgaagcggtgacgacatcggttaaaaaagcagtacgattgaaccgtgcgattcaagcttgggacggtcattggccagccgaaaatgcgggtcccctcttcttcaccccacctctgataattgctctctacataagtggcacattggactcaattctaaccaatgaacacaagaaggaaatgattcgctacatgcacatccatcaaaatgaagacggaggatggggattctatatatcaggaagaagcacaatgatagggagtgctctgaactacgtagctctaaggcttctcggagaagctcctccgccagatggcgacgatggtccacttggtagaggccgcaaatggatacatgatcacggtggcgccacctccatcccatcatggggaaaggtttatctcgcggtgcttggagtatatgaatgggaaggctgcaaccctcttcctccagaattctggctttttccttcctttttgccttatcatccagcaaaaatgtggtgttactgtcgaaccacatacatgccaatgtcacatttgtatggcaagagctaccatggtcccattacagatcttgttttgtctttaagaaaagagattcatcccatcccttaccaccacattaattggaacaaacaacgacataactgttgcaaggaggatctgtactaccctcattcattgatacaagatctactatgggacggtcttcatcattttagcgaacctatcatcagaaaatggccattcaataagctaagagaaaggggtcttaaaagagttgttgacctaatgcgctatggagccgaggaaggacgatatatagccatgggctgcgttgataaggctttacaaataatgagtttttatgcggaggatccaaatgggatcgacttcaaacgacatcttgctagagtccctgattacttgtgggtggcagaggatggtatgaagatgcaaagttttggcagtcaattatgggattgcactcttgtaacacaagcaattatcgcaagtaatatggtagacgagtatggagattcactcaaaaaagctcacttttacttgaaacaatcacagataaaagagaaccctaaaggcgatttcaccaaaatgtgtcgccaattcacgaaaggggcatggactttctctgatcaagatcacggttgggtcgtgtcagattgcactgccgaagctttgaagtgtttattagcactatcacaaatgcctcaagacattgttggagaaaaggctcaagttgatcgattatatgatgctatcaatgtcctcctttacctacaaagtcctgcaaccggtggttttgcaatttgggagcctccggttccaaaaccatatttagagaaattgaatccttcagaactttttgcagatattgttgtcgagaaagagcatgttgaatgtaccgggtctgtaattcaagcgttgcatgcattcaaacatctacatcctgggtaccgtgagaaagaaatagaagttgccattgagaaaggcatacagtttttggaaaacaaacaaaaggatgatgggtcgtggtatggttactggggcatatgttttctctatggcacattcttcgtgctacaaggattggtatcgtgtgggaaaacatatgaaaatagtgaagcaattcggagagctgttcagtttttgctcttgacacggaacttagaaggtggttggggagagagcttcgagtcatgcccaaaagagaaattcatacctttggaggggaatcgaacaaatttggtgcaaacttcatgggccatgctcggtcttctctactgtggacaggctgaaagggatccaacaccattacacaaggcagcaaaactgctcatcaatggacaattggaaaatggagattttcttcaacaggagataacgggatcgtacatgaaaaactgcatgctacattatccggagtataggaacacttttccgttatgggcactcggagaataccgaaagcgtgtttggttgccgaaacaagaagccatctaa

**>*Cp*DDS1 (Cpi03G005455.t1)/*Codonopsis pilosula (Franch.)* (2289bp)**

atgtgtatatccttcagggagtttgatcccgacgccggaacaccagaagagagagaagaggtcgagaaggtccgagaacatttcaggactaacctaagacaaggtgttcatccgtgcagtgatttgctcatgcgcatgcagcttatcaaggagaacagaatagatcttatgagcttaccaccggtgaggcttgcagagaaagaaaatgtgaattatgaagctgttacgatggccgtgaagaaagcacttctattgaaccgtgcaatccaagctaaggatggacactggcctgctgaaaatgctggccctttgttttttacccctccccttatcatcgcattgtacatcagtggaacgattaataccattctacgggaagaacacaagactgagatgattcaatatatttacaaccatcaaaatgaggatggcggatggggattttatattgaaggccatagcactatgatcggttcagcgcttagctatgttgccctgcggttgctgggagaaggtcccgacgatggcaagggtgcagtagggagaggccgaaggtggattcttgatcatgggggtgcaacttcgataccctcttgggggaaagtttatctttcggtgcttggtgtttatgagtggaaaggttgcaatccacttcctccagaattttggttgtttccgtcagctttgccgtatcatcctgcgaagatgtggctttattgtcgaacgagttacttgccaatgacatatttgtatggtaaaagatatcatggaactatcacggatctagttctatcactgagacaagaaattcacaccattgcatacgaggaaatagattggaataaacaacgtcacaactgctgtaaggaagatctctactatcctcatacctttgtacaagatcttctatggaatacacttcacaacgtcagcgagccaattatgacacactggccgttcaagaagatacgagagaaaggcattcgaaaggcagttgagttgatgcgctatggagcacaagagagcagatacatcaccactggatgtgttgaaaagactttacaaatgatgtgctggtgggccgaaaatccaaatggtgacgagttcaaacatcacctcgccagagttccagactacttatggcttgcagaagatggaatgaaaatgcaaagctttggtagtcagttatgggattgttctctagtaactcaagcaatcattgcaactaatatggttgaggactacggcgactcgcttaaaaaggctcactttttcctcaaagaattacaggttaaagaaaatccaacaggagattttaatagtatgtgtcggcattttacaaaaggatcgtggactttttctgaccaggatcatggttatgttgtctccgactgcaccgctgaggcactgaagtgtctactattactgtcacaaatgccagaggagattgctggggcgaaagcggataatgagcgattgtacgaggcagttaacgtccttctatacttgcaaagtcctgaaagcggcggttttgctatttgggagccagcagttcctcaaccatatttacaggttttgaagccttcagaattatttgcagacattgtggttgagaaagagtatggctattggggaatctgcttcctctatgccacattctttgtggtaggagggttaagtcatgctggtcaaacttacaacaatagcaaagccattcgtaaagctatgagcttttttctttcaacacaaaatgaggagggtggttggggtgaaagcattaaatcatgtccaagcgagatatacacaccattggatggaaatcgaacaaatttagtacaaacttcatgggctatgcttggtcttatgtatggaggccaggctgaaagagatccaagacctttacatcgtgcagcaaagttattgattaatgcacaaatggtaaatggagattttccccaacaagaacttactggagtctacatgaaaaattgtatgttgcattacgcagaatatcggaatatttttcccctttgggctcttgcagaatatcgtaaacgtgtttgggtgtccagcaaaaacatgcaatcgaatgatctatggtcagccaaaaaaatctatcatctcggcaatctccttgtttcggccactagcaggttggttttatgtgatatcgtcgatagtttggatttatgtgctatcatcgacgagattcatgatgcgccatcgtcggcaatatttgatgatgcgccttcgtcggcacgtttgatttaccatcttcagcaggattga

**>*Cp*DDS2 (Cpi03G005543.t1)/*Codonopsis pilosula (Franch.)* (2289bp)**

atgtggaaactgaagatagcagaaggtaaaggtccatacttgtatagtaccaacaacttcgttgggagacaaatttgggagtttgatcccaatgcaggaacaccagaagagcgagaagaggtcgagaaggcccgtgaacattttcggattaatcgaagacaaggtattcatgcctgtggtgatttgctcatgcgcatgcagcttatcaaggaaaacggaatagatcttctgagcataccaccggtgaggctcggagagggtgaaaatttgagttatgaagatgttacgattgccgttaagaaagcgcttcgtttgaaccgtgcaatccaagctaacgacggccactggcctgctgaaaatactggccctttgtttttcacccctccctttatcatcgctttttacatcagtggaacaattaataccattctaaggcaagaacacaagaccgagatgatacgatatatttacaaccatcaaaacgatgatggtggatggggattctatattggaggtcacagcactatgatcggttcagcacttagctatgtcaccctacgattgctaggagaaggacccgacgatggcaatggtgcagtggcgagaggccgcaagtggatccttaatcatggaggtgcatgttcgataccgtcctggggaaaaacatatctttcggttcttggtgtttatgagtgggaaggttgcaatccacttcctccagaattctggttgtttcctccagctatgccttatcatcctgcgaagatgtggtgctattgccgaacaacttacatacccatgtcatatttgtacggtaaaaaatatcatgggcccatcacggatctcattctatcactgagaaaagaaattcaccccattgcctacgagagtatagattggaatagacagcgtcacaactgttgtgaggaagatctctactatcctcatacctttgtacaagatcttctatgggatactcttcactaccttagcgagcctataattacacgctggcctttcaagaagatacgagagagaggcattcaaagggcagttgagttgatgcgttatggagcagaagagaccagatacattacacttggatgtgttgaaaagagtttgcaactcatgtgctggtgggcggagaatccaaaaggtaaagagttcaagcatcacctcgctagagttcccgactacttatggcttgcagaagatggtatgaaagtccaaagttttggtagtcaattatgggattgttctctagcaactcaagcaatccttgcaacaaatatggaagaggagtacggcgattctcttaaaagggctaacttctacctgaaagaatcacagattaaagacaatccaactggagactttaacaaaatgtgtcggcagtttacgaaaggatcatggactttctctgaccaagatcagggttgggttgtcggtgactgcaccgctgaggcactaaagtgtctattgttactgtcccaattgccggaggagattgctggagaaaaagccaacactgagcggctttacgaggcagttaatgtcctcctctacttgcaaagtcctggtagcggtggttttggtatttgggagtcaccagttcctcaaccttatttagaggctttaaacccttcggaattatttgcagacattgtggttgagaaagagcatgttgagtgcactgcgtccataatccatgccctggcactcttcaaaagtttgcacccaaagcatcgcaacgaagaaatcgaaatttccgtggaaaaaggaatacactttcttgaacataaacagttccctgatggctcatggtatggttattggggagtctgcttcctctacggcacatttttcgtgttaggagggttagtccatgctggccaatcctatcacgagagcaaagccattcgtaaagctgtaaactttatcctttcaaaacaaaatgaggagggtggttggggtgaaagcatggaatcatgctcaagtgagatatatacaccgttggaaggaaatcggacaaatttaatacagacttcctgggctatgcttggtcttatttatggaggccaggctgaaagagatccaacaccattacataaagcagcaaagttattgattaatgcacaaatggaaaatggagattttccccaacaggaactaaccggagtctacatgagacattgtatgctgcattatgcagaatatcgaaacatttttcccctttgggcactcgcagaatatcacaaacgtgtttggatacccaccataaagttctaa

**>*Cp*DDS3 (Cpi03G005530.t1)/*Codonopsis pilosula (Franch.)* (2289bp)**

atgtggaagctaaagatagccgaaggcaatgatccatacttgtacagcaccaacaactttgttggcaggcaaatttgggagtttgatcccgatgccggaacaccggaggagagggaagaggtcgaaaaagcccgtgaacatttccggagtaatcgaaaacaaggtgttcatccatgtggggatttgctgatgcggatgcaacttatcaaggaaaacggaatagatcttctgagcataccaccggtgaggcttgaagaaggagaaaatttgagttatgaagctgttacgattgccgttaagaaagctcttctattgaaccgagcgatccaagctaaggacggccactggcctgccgaaaactctggccctttgttctttacccctcctcttatcatcgcattgtacatcagtggagcgattaataccattctaaccaaagaacataaggtggagatggttcgatatatttacaaccatcaaaatgatgatggtggatggggattctatattgaaggccatagcactatgcttggttcagcacttagctatgtcgccctacgtttgctaggagaaggtcccgacgatggcaatggtgcagtggctagaggccgaaagtggatccttgatcatgggggtgcaacttcgataccctcttggggaaaaacctatctttcggttcttggtgtatatgagtgggatggttgcaatccacttcctccagaattttggctgtttccttcggctttgccttatcatcctgcgaagatgtggtgttattgtcgaacgacgtacatgccaatgtcgtacttgtatgggaaaaaatatcatgggccgatcacggatcttgttctatcactcagacaagaaattcaccccattgcctacgaggatatagtttggaataaacagcgtcataactgctgtcaggaagatctttactatcctcatacctacgtacaagatcttctatgggatacacttcactatgttagcgagcccatcataacacgctggccgttaaagaagatacgagagagaggcattcaaaaggcagttgagttgatgcgctatggagcacaagagagcagatacattacaattggatgcgttgaaaagagtttacaaatgatgtgctggtgggccgagaatccaaatggtggggagttcaaacgtcatctcgctagagttcccgactacttatggcttgcagaagatggaatgaaaatgcaaagctttggtagtcaattatgggattgtactcttgcaactcaagcaatcattgcaactaatatggttgaggactacggtgactctctaaaaaaggctcacttctacattaaagaatcacaggttaaagaaaatccaagtggagattttagaaaaatgtgccggcagtttacaaagggatcgtggactttttctgaccaagatcaaggttgggttgtctctgactgcactgccgaagcactaaagtgtcttttgttattgtcacaaatgccacaggagattgttggtgtcaaagctgatttggagcagttgtatgaggcagtcaatgtcctcctctacttacaaagtcctgaaagcggtggttttgctatttgggagccaccagttcctcaaccatatttacaggtattgaacccatcggaagtatttgcggacattgtggttgagaaagagcatgttgagtgcactgcgtcaataatccacgccttggtactgtttaaaagtttgcacccaaggcatcgcaaccaagaaattgaaatttctgtggaaaaagggatacgttttctagaggagaaacagttcattgatggctcatggtatggctattggggaatctgcttcctctacggcacattctttgtcgtagcagggttaagtcatgctggccaaacatacaacaatagcaaagccattcgtaaagctgtgaacttttttctatcaacacaaaacgaggaagggggttggggtgaaagcatagaatcatgtccaagcgagatatacacaccattggatggaaatcgaacaaatttagttcaaacctcatgggctatgcttgctcttatgtatggaggccaggctgaaagagatccaacacctttacataaagcagcaaagttacttataaatggacaaatggaaaatggagattttccccaacgggaaattaacggagtctacatgaaaaactgcatgctgcattatgcagaataccgaaacatttttcccttttgggcgctagtagaataccgtaaacgtgtttggatgctgagccaaaagttgtaa

**>*Cr*AS (JQ027033.1)/*Catharanthus roseus* (2289bp)**

atgtggaagctaaagatagcagaaggcaagggaccatacctctatagtacaaacaattttgttgggagacaaatatgggagtatgatcccaacgctggaactcctcaagaaagagaagcattcgaaaaagctcgagaacagttcagaaacaacagaaaaaaaggagttcataatccatgtgctgatttattcatgagaatgcagcttattaaagaaaatggtattgatcttatgagcatcccgccagtaagagttgaagaaaaagaagaattaacttttgaaaaaacgacaattgcagtcaagaaagctctccggctaaaccgtgccattcaagccacagacggccactggcctgcagagaatgccggtcctatgttcttcacaccaccactgttgatcgccttgtatatcagtggagcaattaacacaattctgacatctgaacataagaaggagctggttcgctatatctacaaccatcaaaatgaagatggaggatggggattttatatagaaggtcacagtacaatgattggttcagcacttagttatgtggcattgcgtttgcttggggagggacctgatgatggtgatggtgctgttggcagaggtcggcaatggatacttgatcatggtggtgccactggcattccctcttggggaaaaacttatctttcggttcttggagtatatgattgggatggatgcaacccattgccaccagaattctggttattcccttctttcttcccttatcaccctgcaaaaatgtggtgctactgtcgcacaacctacatgccaatgtcctatttatacggcaaaaaatatcatggcccactcactcatcttgttatgcaactaagacaggaaattcatgtcaagccatatgatcaaattgattggaataaagctcgccatgattgttgcaaggatgacttgtactaccctcattctttcatacaagatcttctttgggacacactcaattatttcagtgagccagttatgagacgttggccttgtaataagatcagagaaaaagctatgagaaaatgcatcaaatatatgcgttatggagcagaagagagcagatatatcactattggatgtgtggagaagagtttgcaaatgatgtgttggtgcgcacacgatccaaattgtgatgaatttaagtatcatcttgctagagttcctgattacttatggctcgcggaggatggaatgaaaatgcagagtttcggaagtcaattgtgggattgtactcttgcaactcaagcaattattgcaactggaatggtggaagaatatggagacactatcaagaaagcacatttctacataaaagaatcacaggtcaaggaaaacccaaaagaagatttcaaagccatgtatcgccactttactaaaggttcatggacattttctgatcaagatcaagggtgggttgtctctgactgtactgctgaagcactcaagtgtttgctggtatgttcacaaatgccacaagatcttgctggagaaaaagctgatgttgagcgcctgtatgatgctgtgaatgttctcctgtatttacaaagtcctgaaagtggtggttttgctatatgggagcctccagttccacagccatatctacaagttttgaatccttcagaactttttgcggacattgttgttgaacaagaacatgttgagaatacagcatctatcgtacaagctcttgtgttattcaaacgtttacatccaggacaccgtgagaaagagatagaagtttctgtctccaaagcagtacgctttctggaaggacgacaatggcctgatggttcatggtatggctactggggaatttgctttctgtatggtaccatgtttgtgctaggaggcctaactgctgctgggaagacatataagaatagtgaagcaatcagaaaagccgttaaattctacttatcaactcaaaatgaagagggtggttggggagaatgtcttgagtcttgccctagcatgaaatacattcctctcgaagggaatcgaacaaatttagtacaaacatcatgggctatgttaggtctcatgtatggagggcaggccgagagagatccaacgccattgcacaaggcagccaagttactaattaatgcccagatggacgatggagacttcccccaacaggaaatcacaggagtttacatgaaaaattgcatgctgcattatgcagagtacagaaatatcttcccattgtgggcacttgcagagtaccgaaaacgtgtctggccaaccaaagctttgtag

**>*Eg*DDS-like (XM_012985994.1)/*Erythranthe guttata* (2334bp)**

atgtggaagctgaagatagctgaaggcaatgggccttacctatacagcacaaacaactttgtaggccgacaaacttgggagtacgatcctaatgccggaacccccgaagaccgcgaatcatttcaaaaggctcgggaccaattcgatcaaaacaggataaagggatttcattcatgtggtgatttgttcatgaggatgcagctcaaaaaggaaagtggaatagatcttgagagcataccagcggttagactcgaagaaaaggaagaaataacttacgaaacagcgacgattgctgtaaagaaagctttgcgattaaatcgagcagttcaagctagcgacggccactggcctgctgaaaatgcagggcctatgcttttcactcccccactccttatagcattgtacataagtggagcaatcaacacaactctgacatcagagcacaaaaaggagatgatccgttacatgtacaaccatcagaatgaggatggagggtggggattatacatagaaggacacagcacaatgatagggtcggcacttagctacgttgcgttacgtatactcggagaaggtgctgacgatggcaacggtgctattgctagagcccgaaaatggattcttgatcatggtggagctactggaattccctcttggggaaaggcctatctttcggtgcttggagtgtatgaatgggaaggttgcaacccacttcccccagagttctggttgttcccttcaattttaccttatcatccagcaaaaatgtggtgctattgtcggacaacttacatgccaatgtcgtacctttacggtagaaaataccacggaccaatcacggaccttgttttatcccttaggaacgaaattcacgtcaagtcctatgatcaaattgattggaataaggcacgccatcattgttgcaaggaagatgtttattacccacacacattcgtacaagatttgctttgggataccctaaactactgcaccgagccagtgatgaagcgttggcctttgaataagattagacaaagagctctcgacaagacgattaaatacatgcgctatggagctgaggagagccgatatattaccatcggatgcgtagaaaagagtctgcagatgatgtgctggtgggcccacgatcagaaatgcgatgagttcaagtatcacctagccagggtccctgattatttgtggctcgccgaagatgggatgacgatgcagagttttgggagtcaaatatgggacagcactctagccacacaagcagttatttcaaccggtatggttgaggaatacggcgactgtcttgagaaggctcacttctacattaaagaatctcaaataaaggaaaatccgaagggcgacttcaggggcatgtatcgtcattttacaaaaggtgcatggactttctcggatcaagatcaaggatgggttgtatcagactgtacagctgaagcactaaagtgcctgcttttgctctcacaaatgccgactgaaatttcgggagaaaaaccgccggttgagcgctcgtacgatgcggtgaacgtgctcctttatttgcagagttctctgagtggtggatttgctatttgggaacctgcaattccacaaccgtatttgcaggttttgaatccctcggaactgtttgctgacatagtcgtcgagcaagagcatgttgaatgcactgcttctatagtccaagctcttatatcgttcaagcgaatgcatccaggccatcgggagaaagaaatcgaaaactctgtggcaagaggagttagcttcctcgaagaaaggcaatggcatgatggttcatggtacggctactggggtatttgcttcctgtacggtacattctttacgctcggaggattagctgcagctggcaaaacatacgaaaactgcgaagccgttaggaaagccgtcaactttttcttgtcttggcaaaatgaagaaggtggatggggagaaagcctcgagtcctgcccaagcatgaaatacacaccactggaaggaaaccgcacaaatttggtgcagacatcatgggcaatgcttggtcttatgtatggtggacaggcggagagggatccgacgcctctgcataaagcagcaaaattgttgattaatgagcagatgagtgatggagatttcccacaacaggaaataaccggagtgtacatgaagaattgcatgcttcattacgcgcagtatcggaatatattcccactgtgggcgctgggagagtatcgtaaacgagtctggccaaaagttattcccaaatcgatctcaacacaacattattcctccctaacttacatatatatttaa

**>*Eg*DDS-like-X1 (XM_012986775.1)/*Erythranthe guttata* (2286bp)**

atgtggaagctgaagatagctgaaggcaatgggccttacctatacagcacaaacaactttgtaggccgacaaacttgggagtacgatcctaatgccggaacccccgaagaccgcgaatcctttcaaaaggctcgagacatattcgatcaaaacaggaaaaagggatttcattcatgtggtgatttgttcatgaggatgcagctcaaaaaggaaagtggaatagatctcgagagcataccaccggttagactcgaagaaaaggaagaaataacttacgaaaccgctactattgctgtgaagaaagctttgcggttaaatcgagcagttcaagctagcgacggccactggcctgccgaaaatgcagggcctatgtttttcactcccccactgcttatagcattgtacataagtggagcaatcaacacaactctgacatcggagcacaaaaaggagatgatccgttacatctacaaccatcagaatgaggatggagggtggggattttacatagaaggacacagcacaatgatagggtcggcacttagctacgttgcattacgtatactcggagaaggtgctgacgatggcaacggtgctattgctagagcccgaaaatggattcttgatcatggtggagctactggaattccctcttgggggaagacctatctttcggtgcttggagtgtatgaatgggaaggttgcaacccacttcccccagagttctggttgttcccttcaattttaccttatcatccagcaaaaatgtggtgctattgtcggacaacttacatgccaatgtcgtacctttacggtagaaaataccacggaccaatcacggaccttgttttatcccttaggaacgaaattcacgtcaagtcctatgatcaaattgattggaataaggcacgccatcattgttgcaaggaagatgtttactacccacacacattcgtacaagatttgctttgggataccctaaactactgcaccgagccggtgatgaagcgttggccgttgaataagattagacaaagagctctcgacaagacgattaagtacatgcgctatggagctgaagagagccgttatattaccatcggatgcgtcgaaaaaagtctgcagatgatgtgctggtgggcccacgatcagaactgcgatgagttcaagtatcacctagccagggtccctgattatttgtggctcgccgaagatgggatgaagatgcagagttttggaagtcaaatatgggacagtactctagccacacaagcagttatttcgactggcatggttgaggaatacggcgactgtcttcagaaggctcacttctacattaaagaatctcagataaaggaaaatccgaagggcgacttcaggggcatgtatcgtcattttacaaaaggtgcatggactttctcggatcaagatcaaggatgggttgtatcagactgtacagctgaagcactaaagtgcctgctgttgctctcacaaatgccggctgaaatttcgggagaaaaaccgccggttgagcgcttgtacgatgcggtgaacgtgctcctttatttgcagagtcctctgagtggtggatttgctatttgggaacccgcaattccgcaaccatatttgcaggttttgaatccctcggaactctttgctgacatagtcgtcgagcaagagcatgttgaatgcactgcttctatagtccaagcccttatatcgttcaagcgtatgcatccaggccatcgggagaaagaaatcgaaaactctgtggcaagaggagttagcttcctcgaagaaaagcaatggcctgatggttcatggtacggctactggggtatttgcttcctgtacggtacattctttacacttggaggattagctgcagctggcaaaacatacgaaaactgcgaagccgttagaaaagccgtcaactttttcttgtcttcgcaaaatgaagaaggtggatggggagaaagcctcgagtcctgcccaagcatgaaatacacaccattggaaggaaaccgcacgaatttggtgcagacatcatgggcaatgcttggtcttatgtatggtggacaggcggagagggatccgatgcctctgcataaagcagcaaaattgttgatcaatgagcagatgattgatggagatttccctcaacaggaaatcaccggagtttacatgaagaactgcatgctccattacgcgcagtataggaatatattcccactgtgggcgctgggagagtatcgtaaacgagtctggccatctcaatccctttaa

**>*Es*DDS1 (Ese18G000975.t1)/*Eleutherococcus senticosus* (2337bp)**

atgtggaagctgaagattgctgagggtcatgggccatatttgtacagcaccaataacttcgttgggagacaaatttgggagtttgtacatgatgcagggactccggaagagcgacaagaagttgaaaaagctcgcgaagccttcacaaagaataaacttagccaaggagttcacccccctggcgatatgctcatgaggatgcagcttatcaaggagagtggaatcgatcttttaagcataccaccagtcagattaggagaaaaggaagaagtgagctacgaagcagcgactacggctgtgaagaaagctcttcgactaaatcgtgcaatccaagcgcatgatggtcactggccagctgaaaatgcaggccctatgttttttacaccaccccttgtcattgccctatacatcagtggagctattaatacaattctaacacaagaacacaagaaggagatgattcgctatatttacaaccaccaaaacaaagatggcggatggggtttctacattgagggccacagcactatgctcggatcagcgcttagctatgttacgttacgtttgctaggagaagggcctgatggtggtaaaggagcagtccaaagagcacgaaaatggatccttgatcaagggggtgcatcttcaataccctcttggggaaagacttatctcgcggttcttggagtatatgagtggggagggtgtaacccattaccccctgagttttggcttttcccttcatttttaccttatcatccagcaaaaatgtggtgctactgccgcacaacttacatgccgatgtcctatttgtatggtaggaaataccatggaccgatcactgatctcgttctatctctaagacaagaaattcaccccatcccatatgaggagataaattggaataaacagcgccataactgttgcaaggaggacctttactaccctcatagctttgtacaagatctgctatgggatggtcttcactaccttagtgaaccaatcatcaagtattggccattcaacaagttgcgacagagaggtctacgtaaagcaattgaacttatgcgctacggagcaacagagagcagatacataactataggatgcgtagagaaaagtttacaaatgatgtgttggtgggcagagaatcctaatggcgatgagttcaaacatcaccttgctagagttcctgattacttatggcttgcagaggatggtatgaaaatgcagagttttggtagtcaagtgtgggactgtactctagcaactcaagcaattatcgcgagtaatatggttgaagaatatggggattctcttaaaaaggcgaatttttacctaaaagaatcacagatgaaagaaaatccatcgggagattttgaaagtatgttccgtcattttactaaaggatcatgggctttctctgatcaagatcatggctgggttgtctctgactgcacagctgaagcactaaagtgtttgctattactttcccaaatgccaacagaaattgcaggagaaaagactaaagttgaacgattgtatgaggcagtaaatgtccttctctacttacaaagtcctgaaagtggtggttttgctatttgggagccaccaattccaaaaccatatttgcagatattgaatccttcagagatttttgccgacattgttgttgagaaagagcatttggagtgcactgcatctataatcgaggctttggtagctttcaaacgtattcaccaatcatatagggagaaagaaataaatacttctgtggaaaaagcagtacattttcttgaaggaaaacaattgccaaatggttcatggtatggctactggggcatttgcttcttgtacggcacatttttcgtgttacgggggttagtttcagttgggaagacatacgataactgtgaagcaattcgcaaagctgttcaatttttcctctcaacacagaatgaagagggtggttggggagagagccttgaatcatgcccaagcgagatatacataccattggatggaaacaggacaaatttagtacaaacatcatgggctatgctaggcctcatgtatggtggacaggctatgagagatccaaccccattacatagagcatcaaagttaattaatgcacaaatggaaaatggagatttccctcaacagaaatcaccaaaaacacgaagcacctacacaattaacagtcaaactaaggaacctgacagcttgggccgtcgagggatagctccgaaaatcgattcggtccggtcctattcagcagattttgcacagtctggttcaacactgtactgcgttcttgaaaagtcttcaacatga

**>*Es*DDS2 (Ese18G000970)/*Eleutherococcus senticosus* (2247bp)**

atgtggaagctgaagattgctgagggtcgtgggccatatttgtacagcaccaataacttcgttgggagacaaatttgggagtttgtacatgatgcagggactccggaagagcgacaagaagttgaaaaagctcgcgaagccttcacaaagaataaacttagccaaggagttcacccctctggcgatatgctcatgaggatgcagcttatcaaggagagtggaatcgatcttttaagcataccaccagtcagattaggagaaaaggaagaagtgagctacgaagcagcgactacggctgtgaagaaagctcttcgactaaatcgtgcaatccaagcgcatgatggtcactggccagctgaaatagcaggccctatgttttttacaccaccccttgtatgtgtttttaatactctcagtagcttagaacacaagaaggagatgattcgctatatttacaaccaccaaaacaaagatggcggatggggtttctacattgagggccacagcactatgatcggatcagcgcttagctatgttacgttacgtttgctaggtgaagggcctgatggtggtaaaggagcagtccaaagagcacgaaaatggatctttgatcaagggggtgcctcttcaataccctcttggggaaagacttatctcgcggttcttggagtatatgagtgggaagggtgtaacccattaccccctgacttttggcttttcccttcatttttaccttttcatccagcaaaaatgtggtgctactgccgcacaacttacatgccgatgtcctatttgtatggtaggaaataccatggaccgatcactgatctcgttctatctctaagacaagaaattcaccccatcccatatgaggagataaattggaataaacagcgccataactgttgcaaggaggacctttactaccctcatagctttgtacaagatctgatatgggatggtcttcactaccttagtgaaccaatcatcaaatattggccattcaacaatttgcgacagagaggtctacgtaaagcaattgaacttatgcgctacggagcaacagagaccagatacataactataggatgcgtagaaaaaagtttacaaatgatgtgttggtgggcagagaatcctaatggcgatgagttcaaacatcaccttgctagagttcctgattacttatggcttgcagaggatggtatgaaaatgcagagttttggtagtcaagtgtgggactgtactctagcaactcaagcaattatcgcgagtaatatggttgaagaatatggggattctcttaaaaaggcgaatttttacctaaaagaatcacagatcaaagaaaatccatcgggagattttgaaagtatgtgccgtctttttactaaaggatcatgggctttctctgatcaagatcatggctgggttgtctctgactgcacagctgaagcactaaagtgtttgctattactttccggaatgccaactgaaattgcaggagaaaagactaaagttgagcgattgtatgaggcagtaaatgtccttctctacttacaaagtcctgaaagtggtggttttgctatttgggagccaccaattccaaaaccatatttgcagatattgaatccttcagagatttttgccgacattgttgttgagaaagagcatttggagtgcactgcatctataatcgaggctttggtagctttcaaacgtattcaccaatcatatagggagaaagaaataaatacttctgtggaaaaagcagtacattttcttgaaggaaaacaatttccaaatggttcatggtatggctactggggcatttgcttcttgtacggcacatttttcgtgttacgggggttagtttcagttgggaagacatatgataactgtgaagcaattcgcaaagctgttcaatttttcctctcaacacagaatgaagagggtggttggggagagagccttgaatcatgcccaagcgagatatacataccattggatggaaaccggacaaatttagtacaaacatcatgggctatgctaggcctcatgtatggtggacaggctatgagagatccaaccccattacatagagcagcaaagttgttaattaatgcacaaatggaaaatggagatttccctcaacagaatacagaaatatatttccattatgggcacttggagaataccgtaaacgtgtttggttgtcgaattgaacgtttgaagtctaagcccgacatgtga

**>*Ha*DDS (XM_022179441.1)/*Helianthus annuus* (2277bp)**

atgtggaagttgaagatagcacaaggggatgatccttacctgtttaccaccaacaattttgttggtaggcaagtttgggaattcgacccaaatggcggaactcaagatgaacgtagagaagttgaagatgcccgtcagcgttttagaaacaatcggagagaaggcatccatccatgtggtgatttgcttatgcggatgcagttgatcaaggaaaaaggaatcgatttattaagcataccgccggtaaggttgggagagaaagaggaaatgaattgtgaagcggcgacgacagcagttaaaaaagcagttagattgaaccgtgcgattcaagcaaaagatggtcattggcctgcagaaaattccggtcctatgtttttcactcctccccttcttattgcaatgtatatcagtggagccatcaacacacatttaaccaaacaacacaagaccgaaatgatacgctatctctacaaccatcaaaatgatgatggagggtggggattttatatcgagggacacagcaccatgttcgggtctgcattaagctacatagccttaaggttgctaggagaaggacccaatgatggagatggtgcggtggagcgaggcaggaagtggatacttaagcatggtggtgcagccacaattccctcttggggcaagacttatctctcggtgcttggtgtatatgaatgggaaggctgcaatccacttccaccagagttttggctttttcctgaagctttaccatatcatccagcaaaaatgtggtgctattgccggacaacttatatgcccatgtcatacttgtacgggagaaaattccatggtccaataactgatcttgttttgcaacttcggcaagaaatccatccgatcccgtatcatgatataaattggaataaacagcgccataactgttgcaaggaagatctctattatcctcactcaacagtccaagatctgttatgggatagccttcattacttgagtgagccaatccttaagtattggccgttcaccaagttaagagaaagaggtctcaaaagagcagttgagctaatgcgatatagtgcccaagagagcagatatattaccattggatgtgtagaaaagagcttgcaaatgatgtgttggtgggctgaaaacccaaatggggatgaattcaagcaccaccttgctagagtgcccgattatttatggctagcagaagatgggatgaaaatgcagagtttcgggagtcaaacatgggattgcacacttgcaactcaagcaataatagcaagtaatatggttgaagaatatggtgattcattaaaaaaggcccatttttatatcaaagaatctcaaatcaaacaaaatccttctggagatttcagtaaaatgtgtcggcagtttactaaagggtcgtggactttctctgaccaagatcaaggctgggttgtctcagattgcacagctgaagcattgaagtgtcttctattactatcgaaaatgcctgaggaaattgtgggagaaaaagcaaaagacgagtggttatatgaggctgttaatgtcctcctttacttgcagagtcctataaccggaggttttgctatttgggagccacctgtcccacaaccatatttacagatgttgaatccttcagaaatttttgcagatattgttgttgagaaagagcatgttgagtgcacagcatcaattatccaagcccttgtagcctttaatcacttgcacccagggtatcgggagaaagaaatagaagtttcagtggcaaaagcggtttgttttttggagggaaagcaattgcaagatggttcatggtacggttattggggaatatgctttctgtatggcacattttttgcactaggaggcttaatttctgctgggaaaacatataacaacagtaaaccagttcgtaaagcagttgagtttttcctttcgaaacaaaatgaagagggtggatggggggagagttacaagtcttgccctagtgaagtatatacaccgttggaagcaaatcgtacgaacctagttcaaacctcatgggctatgcttggtcttatgttaggtggacaggccgaaagagatccgacgccattgcacaaagcagcaaaagtactaattaatgcacagctagataacggggattttcctcaacaggagattactggagtttacatgaagaattgcatgttacattatccagaatacaggaacatcttcccgctttgggcacttggggaatatcgcaaacgagtttggatcaattaa

**>*Ls*DDS-like (XM_023877103.1)/*Lactuca sativa* (2277bp)**

atgtggaagttaaagatagcagaaggaaatgatcattatttgtattccaccaacaattttgttggccggcaaatttgggaatttgaccccgatgctggaactccggtagagcgacaagaagttgaagatgcacgtcagtattttagagacaatcgaagggaaggtgttcatccatgtggcgatctgcttatgcggatgcagttgatcaaagaaaacggaattgatttatttagcataccaccaagaagattgggagtgaatgaggaagtaaattatgatgcagtgacaacatcagttaaaaaagcactccgattaaaccgtgcaattcaagcaaaagatggtcattggcctgcagaaaatgcgggccctatgtttttcactcctccactccttattgctatgtacatcagtggagccatagatacgcatttaaccaaagaacacaaggaagaaatgatacgttatatctacaaccaccaaaatgaagatggagggtggggattttatatagaaggacacagcaccatgattggatctgctttaagctatgtagccctacggttactaggagaaggacccgatgatgggaacggtgcagtgagccgagcaagggagtggatactcgaacatggcggtgcaatgtcgattccttcttggggcaaaacttatctatcggtacttggggtgtatgaatgggatggatgcaatccacttccgccagaattttggcttttcccagaaactttaccatatcatccagcaaaaatgtggtgctattgtcggacaacttatatgcccatgtcatacttgtatgggagaaaattccatggtccaatcactgatcttgttttgcaacttcgacaagaaattcatccgatcccatatgataacataaattggaataaacaacgccacaactgttgcaaggaagatctctactaccctcattcaacacttcaagatttgttgtgggatagtcttaactacttcagcgagccacttcttaagcattggccttttaagaaattaagagaaaaaggtctcaaaagagcagttgaattaatgcgatatagtgctgaagagagtagatatatcactatcggatgtgttgaaaagagcttgcaaatgatgtgttggtgggcagagaatccaaacggggatgaattcaagcatcatcttgctagggttccggattacttatggctagcagaagatggaatgaagatgcaaagtttcgggagccaagtatgggattgtacacttgcaactcaagcaataatcgctagtgatatggttgaagaatatggggattcccttaaaaaagcccatttttatataaaagaatcccaaataaaacaaaacccatctggagattttagtaaaatgtgtcgacagtttactaaaggagcatggactttctctgaccaagatcaaggttgggttgtctcagattgcacagctgaagcacttaagtgtcttttattactatcccaaatgccagaggaaatttcaggagaaaaggctgataatgaaagattatatgaggctgttaatgtccttctttacttacaaagtcctataagtggaggttttgctatttgggagccacctgtccctcaaccatatttacagatgttgaatccttcggagatttttgcagacattgttgttgagaaagagcatgttgagtgcacatcatcaattattcaagcccttttagccttcaaaagattgcacccaggtcatagggagaaagaaattgaaatttctgtggcaaaagcagttggttttttggaggaaaaacaatggcatgatggttcttggtatggttattggggaatatgtttcctatatggcacattttttacaataggaggcttaatttcagctggaaaaacatataacaatagtgaatcggttcgtaaagcagtaaattttttcctttcaacacaaaatgaagagggaggatggggagaaagcatacagtcttgccctagtgaagtatacacaccactggatggaaatcgaacaaatttagttcaaacatcatgggctatgcttggccttatgttatgtggacaggctgaaagagatccaacacccttgcataaagcagcaaagatattgattaatgcacaaatggataatggagattttcctcaacaggagattactggagtctacatgaagaattgcatgctgcattatgcagagtacaggaacattttcccactttgggcacttggggaatatcgcaaacgtgtttgggtcaattaa

**>*Ob*asOSC1 (JQ809437.1)/*Ocimum basilicum* (2298bp)**

atgtggaagctcaagattgctgagggcaacaatccctatttatacagcactaacaacttcgtcggccgacaaatttgggagttcgatcacaatgcgggaacacccgaggaacgagaggctcaacgcgaggcctttcaaaaggcccgcgatgagtggagcgaggggaggaagaagggattccactcgtgcggggatttgttcttgagattgcagcttaaagaggagagtgggatcgatctcctcagtattccgccggtgagggtcggggaagatgaagagattagctatgagaaagcgacgactgcagttaagaaggctctccggttgaaccgtgcggtccaagctagtgatggacactggccggccgaaaatgccggccctatgttcttcactcccccattgctaattgcattgtacattagtggaaccatcaacactgtgttgacgtcggaacacattaaggaattgatccgctacatctacaatcatcagaatgaagacggcgggtggggattttacatcgaagggcatagtacgatgattggatcggcacttagctatgttgcgttacgtatactcggcgaggggcccgacgccggcaacggcgccatggcaagagcaagaaaatggattcttgatcatgaaggagccaccggaattccatcgtggggaaagacttatctttcggttctcggagtgtatgaatgggatggttgcaacccacttcccccagagttctggctcttcccttcaatgtttccttatcatcctgcaaaaatgtggtgttactgccgtacaacgtacatgccaatgtcgtatctatatgccaaacaataccatggacctctcaccgacgtcgttttagccattcgtgatgaaattcacttgaaaccttatgatcagattgattggaacaaggcccgccatcattgttgcaaggaagatctttactatcctcactcagccatccaagatttgttgtgggacactctcaactactgcacggagccggtgatgaggcgttggccgttgaacaagatccggcagaaagccatggacaaggccatcaaatacatgcgttatggagccgaggagagccgatatatcaccatcggatgtgtcgaaaaaattcttcaaatgatgtgttggtgggcccacgacccgaattgcgatgagttcaagcatcatttagctagagtgccggattacttgtggctggccgaagatggaatgaaaatgcagagttttggtagccaactctgggacagtgctcttgcaactcaagcagttatttcaaccggaatggtcgaggaatacggcgactgtttgaagaaggcgcatttctacataaaagagtctcaggtaaaagaaaatccaaaaggagatttcacagccatgtatcgtcacttcacgaaaggttcgtggactttctccgatcaagatcaaggatgggttgtttcagactgcacagccgaagcactcaagtgtttgcttatcctatcacaaatgccgaaagaaatcgcaggcgaaaaggccgatgttgagcgattgtacgaagctgtgaatgtgcttctctacttgcagagtcctctcagtggtggatttgcaatctgggagcctcctgttccacaaccatatctccaagttttgaacccctcagagctattcgcagacatcgtggtcgagcaagagcatgtggaaaacactgcggctataatccaatctctgatagcattcaagcgtctttaccccggccaccgagccaaggaaatcgaagcctctgtatcgaaagccctcagtttcctcgaaggaaaacaatggcccgatggttcatggtatggatattggggaatctgcttcctctatggcacgtgcttcgtgctcggaggcttagcagccgccggaaagacatacgagaacagtgaagcagttcgaaagggtgtcaactttttcttgtcgacacaaaacgaagaaggtggatggggagagagccttgagtcctgcccgagcatgaaatacataccattggaaggaaacagaacaaatctggttcagacatcatgggcaatgctaggtcttatgtatggtggacaggctgagagggatccaaagcctctacacagagcagcaaaattattgatcaatgcacaaatggacgacggagatttccctcaagaggaaataactggtgtttacatgaagaactgcatgctccattacgcgcagtacagaaatgtgtttcctctatgggcgttgggagagtatcgtaaacgagtttggtcatcacaatgcttgtaa

**>*Oe*DDS-like (XM_023036651.1)/*Olea europaea var. sylvestris* (2289bp)**

atgtggaagcttaagattgctgaaggacatggaccatatttgtacagcaccaacaattttgctggcagacaaatttgggagtacgaccctaatggcggaacaccggaagaacgagaagcatatgataaagctcgcgaagaattccagaggaatcggaaattgaagggagttcacccctgtggtgatttattcatgcggattcagcttattaaggaaagtgggatcgatctaatgagcataccgccagttagacttggagaaaaagaagaagtaacctatgaaacagccacgactgccgtgaagaaagctttactcttaaatcgtgcagtacaagcaagcgatggccattggccagctgaaaacgccggtccgatgtttttcactcctccgctgattattgtcttgtatatcagtggagcaattaatactatcctcacatcagaacacagaaaggagatggttcgctacatttacaaccaccagaacgatgatggtggatggggattttacattgaaggtcatagtacgatgattggctctgcacttagttatatagctctacgtttgctgggagaagggcctgacgatggtaatggttcgattgctagagcccgcaaatggatccttgatcatggaggtgcaaccggaattccctcatggggaaagacctatctttcggtccttggagtgtatgattgggatggttgcaacccattaccaccagaattttggcttttcccttcatttttaccttatcatccagccaagatgtggtgttattgtcgtactacatacatgcctatgtcatatttgtacgggagaaaatatcacgggccacttaccgatcttgttttatcgttgagaaacgagattcacatcaagccatataatgagatagactggaataaagcacgtcatgactgttgtaaggaagatttgtactatcctcatagctcaatacaagatcttctttgggatactctcaattactgtgccgagccagtaatgagacgttggccactaaataagattagacaacgagctctaaataagacaatcaaatatatgcgctacggagcagaagagagcagatacattaccatcggatgtgtagaaaagagtctacaaatgatgtgttggtgggcacatgatccaaacggtgacgaatttaagcatcatctcgcaagagttcccgattacttgtggcttgccgaagatggaatgaaaatgcagagttttggtagtcaaatctgggatagtactcttgccactcaagccgttattgcaactggtatggttgaggaatatggggattgccttaagaaggcacacttctatgtgaaagaatctcagataaaagaaaatccggcgggagacttcaaaagtatgtatcgtcactttactaaaggcgcgtggactttctcagatcaagatcaggggtgggttgtatcagactgcacagccgaagcccttaaatgtctcctcttactctcacaactgcccacagaaattgctggagaaaaagcagatgttgagcgcttgtacgaggctgtgaatgtcctcctatatctacaaagtcctgaaagtggtggatttgctatttgggagcccccagttccacagccatatttgcagatgttgaatccttcagaaatttttgcagacatagtggttgaaacagagcacgttgaatgcagtgcttcaataatccaggctcttttggcattcaagcgtctatatccaggccacagggagaaggaaattgaaatttccgtcgctaaagcaatatccttccttgaaggaagacaatggcccgacggctcatggtatggctattggggaatttgcttcttgtatggtacattttttgtgcttggaggattgtctgctgcagggaaaacttatgaaaacagtgaagcagttcgtaaaggagtaaactttttgctatcaacacaaaatgaagaaggtggatggggagaatgccttgaatcatgtccaagcatgaaatacacgcctttggaaggaaatagaacaaatttggtacaaacatcgtgggccatgctaggtcttatgtatggtggacaggcggagagggatccaacatctttacataaagcagcaaagttgttgattaatgcccaaatggatgatggagatttccctcaacaggaaattactggagtatacatgaagaattgtatgttacattacgcacaatacaggaacatttttccattgtgggcacttggagaatatcgtaaacgtgtgtggtcatctcaaagcctgtaa

**>*Ofβ*-AS (KY992862.1)/*Osmanthus fragrans* (2289bp)**

atgtggaagcttaagattgctgaaggacatggaccatatttgtacagcaccaacaattttgctggccgacaaatttgggagtacgaccctaatggcggaacaccagaagaaagagaagcatatgataaagcccgcgaagaattccagaggaatcggaaattgaagggagttcacccctgtggtgatttgttcatgaggattcagcttattaaggaaagtgggatcgatctgatgagcataccgcctgttagactcggagaaaaagaagaagtaacctatgaaacagccacgactgccgtgaagaaagctttacgcttgaatcgtgcagtacaagcaagtgatggccattggccagctgaaaacgccggcccgatgtttttcactcctccactgattattgtcttgtatatcagtggagcaattaatactatcctcacatcagaacacagaaaggagatggttcgctacatttacaaccaccagaacgacgatggtggatggggattttacattgaaggtcatagtacgatgattggctctgcacttagttacatagctctacgtttgctgggagaagggtctgacgatggtaatggtgcgattgctagagcccgcaaatggatccttgatcatggaggtgcaaccggaattccctcatggggaaagacttatctttcggtccttggagtgtatgattgggatggttgcaacccattaccaccagaattctggcttttcccctcatttttaccttatcatccagccaagatgtggtgttattgtcgtactacatacatgcctatgtcatatttgtacgggagaaaataccacgggccacttaccgatctggttttatcgttgagaaacgagattcacgtcaagccgtatgataagatagactggaataaagcacgtcatgactgttgtaaggaagatttgtactatcctcatagcttcatacaagattttctttgggatactctcaattattgcaccgagccggtaatgagacgttggccactgaataagattagacaacgagctctagataagacaatcaaatatttgcgttacggcgcagaagagagcagatacatcaccatcggatgtgtagaaaagagtctacaaatgatgtgttggtgggcacatgatccaaattgtgacgaatttaagcatcacctcgcaagagttcccgattacttgtggcttgccgaagatggaatgaaaatgcagagttttggtagtcaaaattgggatactgctcttgccactcaagccgttatttcaactggtatggttgaagaatatggggattgccttaagaaggcacacttctatgtgaaagagtctcaggtaaaagaaaatccagcaggagacttcaaaagtatgtaccgtcactttactaaaggctcgtggactttctccgatcaagatcaggggtgggtcgtatcagactgcacagccgaagcccttaaatgtctccttttactctcacaaatgcccacagaaattgctggagaaaaagctgatgttgagcgcttgtacgatgctgtgaatgttctcctatatatgcaaagtcctgaaagtggtggatttgctatttgggagcccccagttccacagccatatttgcagatgttgaatccttcagaactttttgcagacatagtagttgaaacagagcacgttgaatgcactgcttcaataatccaggctcttttggcattcaagcgtctacatccaggccacagggagaaggaaattgaaatttccgtcgctaaagcaatacccttccttgaaggaagacaatggcccgatggctcatggtatggctattggggaatttgcttcttgtatggtacattttttgtgcttggaggattgtcttctgcagggaaaacttatgaaaacagtgaagcagttcgtaaaggtgtgaactttttcctaacgacacaaaatgaagaaggtggatggggagaatgccttgaatcatgtccaagcatgaaatacacacctttggaaggaaatagaacaaatttggtacaaacatcgtgggccatgctaggtcttatgtctggtggacaggcggagagggatccaacacctttacataaagcagcaaagttgttgattaatgcccaaatggatgatggaaatttccctcaacaggaaattactggagtatacatgaagaattgtatgttacattacgcacaatataggaatgttttcccattgtgggcactcggagaatatcgtaaacgtgtgtggccatctcaaagcctgtaa

**>*Si*DDS-like (XM_011098260.2)/*Sesamum indicum* (2286bp)**

atgtggaagctgaagattgctgaaggccatggcccatatttgtacagcaccaacaacttcgtcggccgacaaatttgggagtatgatcccaatggcggaacccctgaagaacgccaagcatttcagaaggctcgcgaagaattcaatgagaacaggaagaagggttttcattcttgtgctgatttgttcatgagaatgcagcttaaaagagaaagtggaattgatctgctaagcatcccaccggttagagttggagaaaaggaagaagtgacctacgaggcagcgactattgctgtgaagaaagccctgcgccttaatcgggcagtccaagcaagtgacgggcactggccagctgaaaatgcaggccctatgttttttactcctcccctccttatcgccttgtacataagcggagcaatcaacacaattttgacatccgaacacaagaaggaaatggttcgatacatctacaaccatcagaatgaggacggcggatggggattttacatagaagggcacagcacaatgataggttcagcacttagctatgtggcactacgtattttgggagagggacccgacgacggcaacggtgcagtagcaagggctcgcaagtggattctggatcatggtggtgcaactggaattccctcttggggaaagacctatctttcggtcctgggagtgtacgaatgggatggctgcaacccgcttcccccggagttctggctcttcccttcagttttgccttatcatccagctaaaatgtggtgctactgtcgtacaacttacatgcccatgtcctacctctacggtagaaaataccacggaccacttaccgacttggttctatcaatcagacaagaaatccacgtcaagccctatgatcaaatagattggaataaggcacgccatgactgttgtaaggatgatctttactaccctcacaccttcatacaagatttgctttgggatactctcaactactgcacagaaccatttatgcggcgttggccattaaacaagatcagacaaaaggctatggacaaggcaattaaatacatgcgctatggagctgaggagagcagatatattaccatcggatgtgtagaaaaaagtcttcaaatgatgtgttggtgggcccatgatcctaactgcgacgagttcaagcatcatctagccagggtccctgattatctgtggctcgccgaagatggcatgaagatgcagagttttggaagtcaaatatgggacagtactcttgccacacaagcagttattgctagtggcatggttgacgaatacggcgattgtcttaagaaggcacacttctacgtgaaagaatctcagataaaggaaaacccgaagggcgaccacacagcaatgtatcgtcactttactaaaggtgcgtggactttctctgatcaggatcagggatgggttgtatcagactgcacagctgaggccctcaagtgtttgctcctgctttcacaaatgccaactgaaattgcgggagagaaagccgatgttgagcgcttgtacgaggctgtgaacgtgctcctttatttgcaaagtcctctgagcggtggatttgctatctgggaaccaccaattccgcaaccatacttgcaggtattgaacccctcagaactgtttgcggacattgtcgttgagcaagagcatgttgaatgcactgcttccatagtccaagctcttctagcattcaagaggctgcatccaggccaccgggagaaagaaattgagatctccgtggctaaggcattacagttcctcgaggagaaacaatggcctgatggttcatggtatggatactggggaatttgcttcctctacggcacattcttcgtgctgggaggattagctgctgcagggaagacatacgagaacagcgaagcagttagaaaaggtgtcaactttttcctctcaacacaaaatgaagaaggtggatggggagaaagcttggagtcctgcccaagcatgaaatacacacccttggaaggaaaccgcacaaatttggtacagacatcatgggcaatgctcggtcttttgcaaagtggacaggcagagagggatcctacacctctgcatagagcagcaaagttgttgatcaatgcacagatggacgacggcgacttccctcaacaggaaatcactggagtgtacatgaagaactgtatgctgcattacgcacaatacaggaatatcttcccactgtgggcactgggagagtatcgcaaacgagtctggccatctcaatgcctctaa

**>*Tc*OSC1 (MK351896.1)/*Taraxacum coreanum* (2313bp)**

atgtggaagctgagaataggtgaaaagaatggaaagttcaacattggtgatggaaacggtgatgactatttgcatagcaccaacaactttgtggggagacaaacatgggagttcgaccctgatgcaggcacgcaggaagagcgtgacgaaattgaaaggtttcgagagcaatttttgatcaataaaaagaagcttgacattagctgttgcgcagacttgctcatgcgaaatcagcttattaaggaaagcggaattgatcttataagcgtacctccagtgagacttggagacgacgaagatgtgaactatgaagctgtgacaacagccgtgagaaaggcagttagactgaaccgtgcgattcaagcttgggatggccattggccagctgaaaatgccggtccgctcttcttcactcctcccttgataattgccatgtacataagtggtacattggataccatcctcacacaagaacacaaaagggagatgattcgttatatgtacatccatcaaaatgaagacggagggtgggggttctatatatcaggcagaagcacaatgatagggactgcgctgaattatgtgggtctaagacttcttggagaagatgataatgatgcaattgctaaaggccgtaaatggatacttgatcatggtggcgccacctccattccttcgtggggcaaagtttatatttcggtgcttggagtgtacgaatgggcagggtgtaaccctcttcctccagaattctggctttttccttcatttttgccttatcatccggcaaaaatgtggtgttattgcagaaccacatacatgccaatgtcatacttgtatggtagaggcttccaagggcctattacggaccttgttaagtctttgagaaaagagattcatgtcactccttaccatcagattgattggaacaaacaacgacataattgttgcaaggaggatctttactaccctcatacctacatccaagatctcttatgggatggtcttcattattttagtgagccacttgtatcaaaatggcctttcaagaagttaagagagaagggtttgaaaagagtattggatctaatgcaatataacgccgaggaaggacgttacataactatgggttgcgttgagaaggctttacaaatgatgtgtttttatgcgctagatccgaacggagttgacttcaaacgacaccttgctagactccctgattacttatgggtggcggaggatggtatgaagatgcaaagttttggtagtcagttatgggattgtactcttgtaactcaagcaattatcgctagtgatatggttgaagaatatggagattcacttaaaaaagctaacttttacttaaaagaatcacagatcaaacaaaacccaaaaggcgatttcgaaaacatgtgtcgtcagtttacaaaaggggcgtggactttctctgatcaagatcaaggttgggtcgtctcagattgcactgccgaagctgtgaagtgtttattggcattgtcacaaatgccacaagaaatttccggtgaaaaggtcgacgtcgagcgattatatgatgccattaacgtccttctttacctacagagtccagaaaccggtggttttgcaatttgggaggcaccagttccaaaaccatatttagagaaattgaatccttcagaactttttgcagacatagtggtcgagagagagcatgttgaatgtacggggtccataattcaagcgttgcaaacatttaaaactctgcatccagggcatcgtcaaaaagaaatagaagttgctattgaaaaaggcatacgctttttggaaaatagacaacaagaaaatggttcttggtatggttattggggtatatgttatctttatggaacctatttcgtgttacaaggattggtagcttgtggacaaacatatgaaaatagtgaagcagttcggaaagctgtcaagtttttcctctcgacacaaaactcagaaggtggttggggagagaactttgagtcatgcccacaagagaaatttatacctttggaggggaacagaacaaatttggtgcaaacttcatgggccatgcttggtcttctatgtggtggacaggctgaaagagatccgacaccattacacaaggcagcaaaattgctcataaatggacaaatggataatggagactttcctcaacaggaaataacaggagtgtacatgaagaattgcatgttacattatgcagaatataggaacacttttccgctatgggcgcttggagagtaccgtaagcgtgtttggttaccaaaacaagaaacctaa

**>*Tk*OSC3 (MG646378.1)/*Taraxacum kok-saghyz* (2298bp)**

atgtgggagttaaagatagcggaaggggatggcccttatttgtacagtaccaacaactttgttggtagacaattttgggagtttaatccagatgctggaactccagaagagaaagaggaaatcgaaaaggttcgccagaaatttaaagataatcgtaaaaaaggaggatttcatgcttgtggcgatctactcatgcggatgcagctaatgaaagaaaatgcaatcgatcttacgagcatacttccggtgagaataagtgagggcgagcaagtaaattatgaagcaacaacaattgcagtacgaaaagcagttcgactacatcgtggaatccaagcgaaagatggacattggcctgctgaaaatgctggtcccttgttttttacccctccacttgttattgctttatatatcagtggtaccattaataccgtcttaagtgaagaacacaaaaaagagatgatacgatacttctacaatcatcaaaatgaagatgggggatgggggttctatatcgagggtcatagtacaatgatcggatcagtactgagttatgtagccctacgtctactcggagaaggagaagatgatggagatggtgcaatcgcccgagcacgcaagtggatacttgaccatggtggtgctgcctctattccctcatggggaaaggtttatctttcggtgcttggagtgtacgaatgggagggctgcaatccattgccaccagaattttggctctttccttcgacatttccttttcatcctgctgagatgtggtgctactgccggacaacctacatgcctatgtcatatttatatgggaaaagaatccaaggacctctcacccctctcgtttcctcattgagaaaagaaattcatttaaccccttttgaggatattaattggaataaacaaaggaataattgttgtaagaaggacttctactatccacattcatttcttcaagatgcattgtggcatagccttcactaccttactgagcctgtcctcaagtattggccattttcaaaactacgagggagatctcttgatagagttgtagaactaatgcgctatgaatctgaagagaccagatatatgaccatagggtgtgtcgaaaaaagtctacaaatgatgtgttggtgggcagaaaatcctaatggtgatgagttcaaatatcatctagcaagagttccagattacttatggattgcagaagatggaatgacaatgcatagtttcggtagtcaagtgtgggattgttctcttgcaactcaagcaattatagcaagtaacatggtagaggaatatgatgattgtctggaaaaggcacacttctatttaagagaatcacaggtaaaagaaaatccttcaggagatttcactcgcatgtgtcggcagttcactaagggatcgtggaccttctctgatcaagatcatggatggacagtctctgactgtacagctgaagcactaaagtgtctattgttactatcaaacatgcctaaaaatatcgctggagagaaagatgacactgccagactatatgaagcagttaatgtgcttctttacatgcaaagtcctgtaagtgggggatttgctgtttgggagccaccaattccaaaaccgtttctacagcttcttaatccttcagagatttttgcagatatcgtcgttgagaaagagcatgtggagacaacatcttccattatcggagctctgatcgagttcaaacgggtccatccaaaccacagaaaagaagaaatagaatactcaatctcgaatggaatacgttatctcgaggaaacacaatggcatgatggttcatggtatggttattggggagtatgcttcatatatggaaccttctttgctctgagagcattaagtactgccggaaagacatataagaataacgaagcagcctgtaaaggtgtcaaattcttattgtcgatacaaaatgaagaggggggttggggagagagcctactatcttgccctactgaggtatatacaccgttggatggaaatcagacaaatttggtgcaaacatcgtgggctatgcttggcctactttttggtgggcaggtggatagagatcccacgcctttacatagagcagcaaagctgttgattaatgctcagatggataatggagattttcctcaacaggaaattaccggagtctacatgaagaattgcttgctgctttacgcacaatacagaaacatttttccactatgggcacttggggaataccgtaaacgtgtttggagcataaaaaagggtattcaaaattaa

**>*Pg*OSCPNY1 (AB009030)/ *Panax ginseng* (2292bp)**

atgtggaagcttaagatagcggaagggaataagaatgacccgtatttgtacagtaccaataattttgtagggcggcaaacgtgggagttcgacccggattatgtggctagtcccggagagctagaggaggtggagcaagttcgacgtcagttttgggataaccggtatcaggtcaagcctagtggtgatctcctctggcgtatgcagttcctaagagagaagaatttcagacaaacaatcccccaagtgaaggtaggagatgacgaggcagttacttatgaggccgctaccaccacactccgaagggccgtccactttttttcagctttgcaggccagcgacggtcattggcctgcagagaattccggacctctctttttccttccgcccttggtgatgtgtgtatacattacagggcatcttgatacagtgtttcctgcagaacatcgaaaagaaattcttcgctacatatactgtcatcagaatgaagatggcgggtggggactccatattgaggggcatagcaccatgttctgcacaactcttagctacatttgtatgcgtatacttggagaagggcccgatggtggtgtaaacaatgcatgtgccagaggccgaaaatggatccttgaccatggcagtgtaaccgctataccttcatggggcaagacttggctttcgatacttggtgtatatgaatggataggaagcaacccaatgcccccagaattctggattctcccttctttccttcctatgcacccagctaaaatgtggtgttattgccggatggtttacatgccaatgtcatatttatatgggaagaggtttgttggtccaatcactcctcttatcttacaattaagagaagaactttatggtcaaccctacaatgaaatcaattggagaaaaacacgtcgtgtgtgtgccaaggaggacatctactatcctcatcctttaatacaagacctgctctgggatagtctctatgtattaactgaaccacttttaactcgttggccatttaacaagttgagagagaaagctctgcagacaaccatgaaacacattcactatgaagatgagaacagtcgatatattaccattggatgtgtggaaaaggttttgtgtatgcttgtttgttgggttgaggatccaaatggagattatttcaggaaacaccttgcaaggatcccagattatatatgggttgctgaagatggaatgaagatgcagagttttggtagtcaggaatgggatacaggtttcagcattcaagcattgttggatagtgatctaactcatgaaattggacctactctaatgaaaggacacgacttcatcaaaaagtcccaggtcaaggataatccttctggtgactttaaaagcatgtatcgtcacatttcgaaaggatcgtggactttttcagatcaagatcacggatggcaagtttctgattgcactgcagaaggactaaagtgttgccttattttctcaacgatgccagaggaaatagttggcaagaaaataaaaccagaaagactgtatgattctgttaatgtgttgctttctctacagagaaaaaatggtggcttatcagcatgggagcctgcaggagctcaggaatggttggagttgctcaatcctacagaattctttgcagacattgtcatcgagcacgagtatgtagagtgcacttcatcggcaatccaagctctggttctgtttaaaaagttatatcctggacaccgaaagaaggagatagataattttattacgaatgctgtccgttaccttgaagacacacaaatgcctgatggttcatggtatggaaactggggtgtgtgctttacttatggtagctggtttgctcttggggggctagcggcagctggaaagacatactacaattgtgcagctgttcgtaaagctgttgaatttcttctcaaatcacagatggatgatggcggttggggagaaagctacctttcttgtccgaaaaaggtatatgtaccattagaaggaaaccgctcaaatttggtacatacaggatgggccttgatgggactcattcattctgagcaggctgagagagacccaacacctcttcaccgtgcagccaagttattgatcaattctcagatggaagatggtgattttccccaacaggaaataagtggagtttttatgaagaattgcatgttgcactatgcagcttaccggaatatatacccattgtgggctctagcagagtatcggaggcgggtcccattaccgtccctaggcacctaa

**>*Et*AS (AB206469)/*Euphorbia tirucalli* (2289bp)**

atgtggaagctgaagatagcagaggggggaaatgatgaatatttgtacagtacaaacaattacgtggggagacagacttgggtatttgatccacagcctcctactcctcaagagcttgctcaggttcaacaagctcgtcttaatttctacaataatcgctatcatgtcaagcccagttctgatcttctctggcgtttccagtttctgagagagaagaatttcaagcaaacaataccacaagcgaagattaacgagggagaggacataacatacgaaaaagcaacaacagcattaagaagggcagtccacttcttctcggcgttgcaggctagtgatggacattggcctgctgaaaatgctggccctttgtttttcctcccaccacttgtaatgtgtttgtacataacaggacacttggacactgtgtttcctgctccccatcgccttgaaatccttcgttacatctactgtcatcagaatgaagatggaggatgggggctgcacatagagggacacagcaccatgttctgcactgtgctaagttacatttgtatgcgtcttctgggagaaggtcctaatggcggtcaagacaatgcctgttctagagctcgaaaatggattattgaccatggcggtgccacttacattccctcttggggaaaaacttggctttcgattcttggtgtttatgaatggtccggaagcaaccctatgcctcctgaattttggattcttcctacctttttacctatgcatccagcaaaaatgtggtgttactgtcggatggtttacatgccaatgtcttacctgtatggaaaaagatttgtcggaccaattacacctctcattctccagttgaggcaagaactccatactcagccctaccatcacatcaattggaccaaaactcgtcatctctgtgcccatgaagatgtgtattacccacatcctttgattcaagatttgatgtgggactctctatacatattcacggagcctttacttacacgttggcctttcaacaaaatcataagaaagaaagcacttgaagtcacaatgaagcacattcattatgaagatgaaaacagccgttacattaccattggatgtgttgaaaaggtattgtgtatgcttgcttgttgggctgaagatccaaatggagttcccttcaagaaacatctagccaggatccctgattacatgtgggttgctgaagatggcatgaaaatgcagagctttgggagtcagcagtgggacacaggttttgcgattcaggcactgctagcaagcaatctaacagaagaaataggacaagtactgaagaaaggacatgatttcataaagaaatcacaggtgaaggaaaatccatcaggtgactttaagagcatgcataggcatatatccaaaggatcatggactttctctgatcaggatcatggttggcaagtttctgattgcactgctgaaggattaaagtgctgcctacttttttcaatgatgccaccagaaattgttggtgagaaaatggatgctcaacacttgtacaatgctgtcaacatccttatctctctacagagcaaaaatggaggtttagcagcatgggagcctgctggggctcaacaatggttggaaatgctgaatcccacagaattcttcgcagacatagtgatagaacatgaatatgtagagtgcacagcatctgcaatccatgcattgatcatgtttaagaaattatatccaggccatagaaagaaggagattgagaattttataacgaatgcagtcaaatacctggaagatgtacaaactgcagatgggggatggtatggaaactggggagtttgcttcacgtatgggacatggtttgcggtgggtggactggcagctgctggaaagaattataacaattgtgcagctatgcgcaaagctgttgattttcttctcagaactcaaaaacaagatggaggttggggagaaagctacctttcctgcccccacaagaaatatgtacctctcgaagacaatcgatccaatttagtgcatacttcttgggcattgatgggcctaatttcagctgggcagatggatagagaccctacacctcttcaccgggctgcaaagctattaatcaactcccaattggaagatggtgattttcctcagcaggagataacgggtgtgttcatgaagaattgcatgttacactatgcagcatacagaaacatatacccattatgggctctggctgaataccggaatagagttccattgccttccactactctttaa

**>*Gs*AS1 (FJ790411)/*Gentiana straminea* (2268bp)**

atgtggaggctaaaaatcggtgaaggtggtaatgacccaaatctctacagtactaacaactatgtcggaagacagatatgggagtacgacaggaatgccggaatcgctgaggaaagggaagaggttgaagaagctcgccgcaatttctggaacaaccgccgccatgttaagcccagcagtgatatcctttggcgcttgcagtttttgcgtgaaaagaatttcaagcagagcataccagcagtgaaggtagacgatggccaggaaattaaccaccaaatggccaccactgctttacgcagagctgtccacctcctttcagctttgcagtcttccgacggccattggccggccgaaaatgccggctctttgttctttcttcctcctctgattatatgcttgtacatcacaggccatctgaacaatatatttccaacggagcatacacacgaaattctccgttacctgtactgccatcagaatgaggatggtgggtggggattgcacatagagggtcatagcactatgttctgcacagctttgaattacatttgcatgagaatactcggagaaggacctgatggagggaaggaaaacgcgtgtgctagggcaaggaaatggattcttgaccatggtagtgtcactgcaattccttctctgggaaaggcatggctttcgattcttggactatatgactggtcagggaccaatcccatgcccccagaaatttggagtctgccatcttctctttctatccatccaggaaaaatggggtgctactgtagaatggtttacatgccgatgtcatatttgtacgggaaaagatttgtttgtcgaattacacctttaatttcagaactgagagaggagttacactgtgagccatataatcaaattcgttggaaaagatatagacatgtttgtgcaaaggaggatctatattatcctcatccccttatacaagatttgatatgggattttttttatatatgcactgagccactttttactcactggcctgtaaataagctgagagcaaaagctctagatgccacaatgaaacatatacattatgaagatgaaaatagtcgctatatcaccgttggatctgttgagaaggtgttatgtatgcttgcttgttgggttgaggatcctaatggggattacttccagaaacacctagcgagaatccccgactacatatgggttgcagaagatggaatgaagatgcagagttttggtagtcaactgtgggacatatgctttgccattcaggcacttcttgctagtgatatgactgaagaaatcacagatactctgcgtaagggacacgacttcataaaaaactctcaggtaaaggaaaacccttccggtgacttcaaaagcatgtaccgtcacatgtccaaaggttcatggactttctcagatcaagatcatggatggcaggtctctgattgcactgcagaaggtttaaagtgctgtctcctgttatctacaatgcctcaagagattgttggtgagagaattgaactcgaacgaatctatgacgccgtcaacatattactatccttgcagagcaaaaatggtggccttcctggatgggaaccagtaagggcatcagattggcttgaactgctcaatccaaccgactcctttgccgaccttgtcattgagcatgagcatgccgagtgtactgcatctgcaatcgacgctcttgttttattcaagaaactgtaccccaatcacatgactaaagagattgagagtttcatcacaaatgcttccaactatttagaagatgtccaaatgcctgacggctcatggtatggaaattggggagtgtgcttcatatatggtacctggtttgctcttagaggattagcagcatcaggaaagacatacaataattgccaagttgttcgtaaggccgttaactttttgctaacaacgcaacagaaagatggtggttggggagaaagctatcgttcttgtcctgaaatgaaatatataccgctagaaggaaatcgctcgaacttggtccatactgcttgggctatgatgggtctaattcattccggacaagccaaaagagatcccacgccgctccattgtgcagcaaaactgttaatcaattctcagctggaaaatggagattttccccaacaggaaatcgctggagttttcttcaagaacggcatgctacattttgcttcatacaggtctatatatcccttgtgggctttagcagaataccgcaagcaagtgccgtag

**>*Gs*AS2 (KJ467352)/*Gentiana straminea* (2277bp)**

atgtggaggctgaagatcgcagaaggtggtaatgatccttatttgtatagtacaaacaactatgtagggaggcagacatgggagtacgacccgaacgctggtacgccggaggagagggcggaggtggaggaagctcgccgtcagttctgggacaaccgccgtgatgttaagcctagtggtgatctcctctggcgtttgcagtttttacgtgaaaagaatttcaaacaaaccataccagctgtgaaagtagaagatggccaagaaattactcaccaaatggccaccgattctttacgcagagccgtccatttcttctccgccttgcaggcctccgacggccattggccggccgaaaatgccggccctttgttctttcttccacctctggtgatgtgtctatacatcacaggtcatctcaactcagtatttccagcagaacataaacgtgaaatactacgttacatatattgtcatcagaatgaagatgggggttggggattacacatagaaggtcacagcacgatgttttgcactgcattgagttacatttgcatgaggctactcggagaagggcccgatggtgggattaataacgcgtgtgctagagcacggaaatggattcttgatcatgggagcgtcacttcgattccctcttggggaaagacttggctttcgattcttggagtatacgagtggtccgggactaatccgatgcccccggaattttggattcttccttcttttcttcctatgtatccagcgaaaatgtggtgctattgcagaatggtttacatgccgatgtcgtatttatatggtaaaagattcgtgggcccgattacgcccttaattcgagaactccgagacgagttacactctscgccctatgatcaagtttcttggaaaaaatacagacatgtttgtgcaaaggaagatctgtattaccctcatccatttgtacaagatttgatttgggatagtttatatgtatgcacagagccacttttgactagatggccattcaacaaattgagagcaaaagctcttgaagtaacaatggaacatattcattatgaagatgaaaatagtcgttacataactattggatgtgttgaaaaggtgttatgtatgcttgcttgttgggttgaggatccaaaaggggattacttcaagaaacatcttgcaagaatcccggactatatatgggttgcagaagatggaatgaaaatgcagagttttggtagtcaagaatgggatacaggttttgccattcaagcacttcttgcgtgtgatatgaccgaagaaatcgcagaaactctacgtaaaggacacgactttgtgaagaaatcccaggtcaaagataacccttctggtgattttaagagcatgcaccgccacatttcgaaaggttcatggacattttcggatcaagatcatggatggcaagtctctgattgcaccgctgaaggcttaaaggtttgtctccatttttctacaatgccaccagagattgttggtgagaaaatggagcctgagaaaatctatgacgctgtcaacattttattaccgttacagagcaaaaatggaggcctatcagcatgggagccagcagggtcatcagaatggctagagctgttgaatccaactgaattctttgctgatattgtgattgagcatgagtatgttgaatgtactgcatcttcaatccatgctctcattttattcaagaaactctacccgggacaccgtaagaaagaaatcgaaacgtccattataaacgcgtcgaaatatctagaagatgtccaaatgccagatggctcatggtatggaaattggggagtgtgttttacatatggtacatggtttgcacttggaggattagcagcatctggaaagacatatgataattgtaacgcggttcgtaaagctgttaatttcttgctaaattcacaacgacaagatggcggttggggtgaaagctatctttcttgtcctcaaaagatatacgtacctcttgaaggcgaacgctcaaacttggttcatacttcttgggctatgatgggactaattcattctggacaggccgaacgagatcctacgcctctccatcgtgcagctaaacttataatcaactctcagatggaagatggagattttccccaacaggaaataacaggagttttcatgaagaattgtatgttacattatgctgcgtatcggaacatatatcctttatgggctttagcagaatatcgcaagagagtgccattgccatcttga

**>*Ob*asOSC1 (KF636411)/*Ocimum basilicum* (2286bp)**

atgtggaggctgaagattgcggaaggcgtggacggcccttatctctacagcaccaacaactacgtcggccgccaaacttgggaatttgatcccgactatggcacgccggacctccgccaacagatcgaggacgcccgccgcgatttctgggacaaccgccaccaggtcaagccaagtagcgacgtcctctggcgtaaacagttcttgcatgagaagaatttcaagcacacaatcccccaagtgaaagtgggcgacggcgaagaaataactcatgaggccgccactgccactctccggcgagccgtacacttcttctccgctttgcaagcaagcgacggccactggcccgccgagaatgccggccctatgttcttccttccacctctcgtgatgattacgtacattaccggccatctgaacacggtgtttccggcggagcatcgtcgtgaaatcctgcgttacacgtattgtcatcagaatgaagatggtggatggggattccacatagaagggcacagcacgatgttttgcacagtgttgaattatatatgcatgcgcattttacgcgaaagcccagatggagggctaaacaatgcctgcagcagagcaagaaaatggattcttgatcatggcactgccaaagccattccttcatggggaaagacttggctttcgatattgggagtttttgattggtcaggaagcaacccaatgccccctgagttttggctacttccttctttcctccctatgcatccagcaaaaatgtggtgctactgtcgtatggtgtacatgccaatgtcatatctatatggaaagagatttgtaggcccaattactcctcttgtcaaagagctcagagaagagctctatgatcagccttatgaagaaatcaattggagaagcattagacatctatgtgccaaggaggatctctactaccctcatcccctgatccaagacttgatctgggacagctgttacgtattaaccgagcctcttctaacccgatggcctttcaacaagttaagagagaaagcactcaaagtgacaatggagcacatccactacgaagacgaaaacagccgatacatcaccattggatgcgtcgagaaggtattgtgcatgctggcatgctgggttgaagatcccaatggcgactacttcaagaaacatcttgcaagaatcccggattatctatgggttgcagaagatggaatgaagatgcagagcttcggtagtcaagaatgggacacgggtttcgccatacaagctctgttagccagtgacatggctgatgaaataggcgaaactctcaagagagggcatgagttcatcaagaattctcaggtaaaggaggatccttctggagacttcagaggcatgtaccgacatatctcgaaagggtcgtggacgttttcagaccaagatcatggatggcaagtgtctgattgtactgcagaaggattgaagtgttgccttctcttctccatgatgaatcctgaaattgttggagaaaaaatggaacctgagggactctataatgctgtcaacatattaatttccttgcagagcaagaatggtggcttagcagcttgggagccagcaggagcctcagagtggctagagttgctgaatcctacggagttttttgctgacatagtcattgagcatgaatatgtggagtgcacttcatcagcaatccaagcacttgttctgttcaagaagctgcaccctcatcaccgaaagaaggagattgaaaccacgatcgaaaaggccgctggatacctcgaggatgtccaacggcccgacggctcatggtacggaaactggggagtttgcttcacttatggcacgtggttcgccctaggagggctggctgcagccggccggacctacaacaactgcgccgcggttaggaaaggtgtcgattttcttcttaaatcacaaagggaggatggtgggtggggagagagctacctctcctgccccaagaaggaatacgtcccactagaacatgatcggtcgaacttggtgcacacttcatgggccatgatgggtctaatccattcgggccagagtgagagggaccctacacctctccaccgtggagctaagcttcttatcaattctcaattggaagatggtgattttccgcaacaggaaattactggagttttcatgaagaattgcatgttgcattatgcagcatacagaaacatatacccactttgggctctagctgagtaccgaaagcgtgttacgcttccatcaagagcaacataa

**>*Tw*OSC2 (KY885468)/*Tripterygium wilfordii* (2292bp)**

atgtggaggctaaagattgcagagggagggaatgaggctgagccatacttatacagcacaaacaattatgttggtagacaaacatgggagtttgatcctaattctggcactcctgaggagcgagctctggtcgaaaaagctcgcgacgatttctaccagaatcgctatcatgtcaagcctagtagcgatctcctctggcgaatgcagttcctgcaagaaaaaaatttcaagcaaacaataccagctgtcaaggtagaggatggtgaagaaatcacacatgaaatagccacaactgccttaagaagggctgtccacttctttggggccttgcaggcccacgatggccactggcccgctgaaaatgccggcccattgttcttcctccctcccctggtgatgtgcatgtacatcacaggacatcttgatgctgtatttcctgaagaacatcgcacagaaatccttcgttacatatataatcatcagaatgaagatggtggatggggattacacatagaaggtcacagcacaatgttctgcacagttcttagctatgtatgtatgcgtattctcggtgaaggaccggacggtggccggaataatgcctgtggtagagcaagaaaatggattcatgaccatggtggtgtcacttacataccttcttgggggaagacttggctttcaattattgggttgtttgattggtgtggaaccaacccaatgcctccagagttttggatcctcccttcatttcttcctatgcatccagcaaaaatgtggtgttattgccggatggtgtatatgccaatgtcatatctctacggcaagagatttgttggtccaattacacctctcatcttacaactcagagatgaactccatactcaaccctacaaccagattaattggaagaaagttcgccatttatgtgctaaggaagatctgtactatcctcatcctttgatacaagacttgttatgggatagtctgtacatagtttccgagcctcttctgacgcgctggccgctaaataagatggttagagagcatgctctaaaagtaacaatggagcacatccattatgaagatgagaacagcagatatataacaataggatgtgtagagaaggttctatgtatgcttgcttgttgggttgaagacccaaatggggattatttcaaaaaacatcttgctaggatcccagattacttgtgggttgctgaagatggaatgaaaatgcaaagttttggtagccaagagtgggatgctggatttgccattcaagctttgcttgcttgcaatctcgtagatgaaattggagatgtgcttgcaagaggacatgactttataaaggcttctcagatcaaagacaatccttctggtgacttcaagagcatgcacagacatatttctaaggggtcttggactttctctgaccaagaccatggatggcaagtttcagattgcactgctgaaggtttgaagtgttgtttgcttttctcaatgatgtcaccagaaattgttggtgagaaaatggagcctgagagattatatgatgctgttaatatcttgctgtctcttcaaagtaaaaatggaggtttagcagcttgggaacctgcaggggctcaagagtggctagaattgctgaatccgacggagtttttcgcggacattgttgttgaacacgaatacgttgagtgcacctcatctgccattcaagcttttgtattgttcaagaagttgtaccctggatataggaagaaagagattgaagtctccattgaaaatgctgcaaagtaccttgaaaatgtgcaaatgcctgatggttcatggtatgggaactggggagtttgcttcacatatgggacctggtttgcacttggagggctgacagccactggtaggacatacaacaactgtgctgctacaagaaaagccgtcgattttcttatcagaacacagagagacgacgggggctggggcgagagttacctttcatgtccagagaagagatacgtgcccctcgaaggaggcggatcgaacctggtacatacagcatgggctatgatgggactaattcatgctgaccaggcagaaagagacccgacgcctcttcatcgtgccgcgaagttgataatcaattctcaactggagagtggtgatttcccacaacaggaaatcacaggtgttttcatgaagaactgcatgttacactatgcagcatacagaaacatttatccactgtgggcattagcagagtatcggaagagagtaacattatcttccaaagtctag
